# The role of ROCK1/MLC/NMMHC IIA-actin signaling in ischemic stroke-induced blood-brain barrier disruption: implications for therapeutic intervention

**DOI:** 10.1007/s00018-025-05808-4

**Published:** 2025-10-30

**Authors:** Liangying Bao, Yuanhao Xu, Yuchuan Ren, Yujie Dai, Junhe Yu, Milin Zhang, Shuaishuai Gong, Junping Kou

**Affiliations:** State Key Laboratory of Natural Medicines, Jiangsu Key Laboratory of TCM Evaluation and Translational Research, Department of Pharmacology of Chinese Materia Medica, School of Traditional Chinese Pharmacy, China Pharmaceutical University, Nanjing, 211198 People’s Republic of China

**Keywords:** ROCK1/MLC/NMMHC IIA-actin loop, Ischemic stroke, Blood-brain barrier disruption, Tight junction

## Abstract

**Background:**

Disruption of the blood-brain barrier (BBB) is a key event in the onset of ischemic stroke (IS), primarily driven by endothelial cytoskeletal rearrangement. The interaction between non-muscle myosin heavy chain IIA (NMMHC IIA) and actin, along with the ROCK/MLC pathway, is central to this cytoskeletal reorganization. While our previous studies have shown that the Caspase-3/ROCK1/MLC/NMMHC IIA-actin positive feedback loop mediates H_2_O_2_-induced neuronal apoptosis, its role in cerebral ischemia-reperfusion (I/R) injury and BBB disruption remains unclear.

**Methods:**

In vivo, we used endothelial-specific NMMHC IIA conditional knockdown mice, NMMHC IIA-inducible endothelial conditional knock-in mice and C57BL/6J to establish a middle cerebral artery occlusion/reperfusion model. In vitro, we employed brain microvascular endothelial cells in an oxygen-glucose deprivation/reoxygenation model. The effects of the NMMHC IIA inhibitor blebbistatin, the ROCK1 inhibitor Y-27632, and the actin depolymerizer cytochalasin D were assessed for their impact on I/R-induced activation of the ROCK/MLC/NMMHC IIA-actin pathway, tight junction proteins (TJs) degradation, and brain damage.

**Results:**

Inhibition of NMMHC IIA expression and stress fiber depolymerization significantly reduced NMMHC IIA-actin interactions, suppressed the ROCK/MLC pathway, decreased TJs degradation, and alleviated cerebral I/R injury. Conversely, overexpression of NMMHC IIA further exacerbated cerebral I/R injury and BBB disruption and amplified activation of the ROCK1/MLC pathway. Y-27632 inhibited the ROCK/MLC/NMMHC IIA-actin pathway, mitigating I/R-induced BBB disruption.

**Conclusions:**

This study reveals that the ROCK1/MLC/NMMHC IIA-actin pathway is implicated in I/R-induced BBB disruption and operates as a positive feedback loop. These findings offer a promising therapeutic strategy for the treatment of IS and BBB damage.

**Supplementary Information:**

The online version contains supplementary material available at 10.1007/s00018-025-05808-4.

## Introduction

Ischemic stroke (IS) remains a leading cause of disability and mortality worldwide, affecting millions of individuals annually [1]. Current clinical interventions include rt-PA thrombolysis and mechanical thrombectomy [2], yet these therapies are constrained by a narrow therapeutic window (4–5 h) and often precipitate cerebral ischemia-reperfusion (I/R) injury, aggravating the initial brain damage. Consequently, fewer than 3% of patients benefit from these treatments [3–5]. Thus, there is an urgent need to identify novel therapeutic targets and pharmacological agents for ischemic stroke.

Among the various pathological mechanisms of stroke, disruption of the blood-brain barrier (BBB) is a key contributor to cerebral I/R injury, instigating a cascade of processes such as inflammation, neovascularization, apoptosis, and necrosis [5–7]. Identifying interventions to preserve BBB integrity during ischemic pathology is therefore crucial. Notably, early BBB disruption has been linked to endothelial cytoskeletal rearrangement [8]. As a key component of the cytoskeleton, non-muscle myosin IIA (NMMHC IIA) interacts with actin filaments to form stress fibers under pathological conditions, exerting contractile forces that destabilize endothelial cell junctions and increase BBB permeability [9, 10]. Specific knockdown of endothelial NMMHC IIA markedly reduces BBB permeability and mitigates I/R injury [11]. Additionally, the NMMHC IIA-actin-Atg9 interaction has been implicated in modulating cerebral ischemia by regulating neuronal autophagy [12].

It has been demonstrated that the RhoA/ROCK1 signaling pathway phosphorylates myosin light chain (MLC), thereby activating the ATPase at the NMMHC IIA head, which provides the energy required for endothelial cytoskeletal rearrangement and subsequent BBB disruption [13–15]. Our previous research revealed that the interaction between NMMHC IIA and actin mediates oxidative stress-induced neuronal apoptosis, further amplifying actomyosin contractility via a Caspase-3/ROCK1/MLC signaling feedback loop [16]. However, it remains unclear whether this positive feedback mechanism contributes to BBB disruption during cerebral I/R injury.

To investigate these issues, we adopted middle cerebral artery occlusion/reperfusion (MCAO/R) models in C57BL/6J mice, NMMHC IIA endothelial conditional knockdown (*Myh9*^*ECKD*^) mice and NMMHC IIA-inducible endothelial conditional knock-in (*Myh9*^*iECKI*^) mice, alongside oxygen-glucose deprivation/re-oxygenation (OGD/R) models in cerebral microvascular endothelial cells. In each experimental group, NMMHC IIA inhibitor blebbistatin, ROCK1 inhibitor Y-27632, and F-actin depolymerizing agent cytochalasin D were administered to elucidate the role and underlying mechanisms of the ROCK/MLC/NMMHC IIA-actin loop in cerebral I/R and BBB integrity.

Our findings demonstrated that Y-27632 effectively inhibited ROCK/MLC pathway, the NMMHC IIA-actin interaction, ischemic damage, and BBB disruption. Furthermore, suppression of NMMHC IIA expression or the NMMHC IIA-actin interaction ameliorated I/R injury and preserved BBB integrity by inhibiting the ROCK/MLC pathway. Conversely, overexpression of NMMHC IIA further exacerbated I/R-induced BBB disruption and amplified activation of the ROCK1/MLC pathway. These results strongly support the existence of a positive feedback loop between NMMHC IIA-actin and the ROCK/MLC signaling pathway in ischemic stroke and BBB disruption, providing a promising strategy for therapeutic intervention in cerebral ischemia.

## Materials and methods

### Animals

Male *Myh9*^*ECKD*^ and *Myh9*^*iECKI*^ mice (SPF grade, 18–20 g), were supplied by the Model Animal Research Institute of Nanjing University (license number: SCXK 2018-0008; Nanjing, China). Briefly, following our established protocol [17], *Myh9*^*ECKD*^ mice were generated by crossing of *Myh9*^*fl/fl*^ mice with *Cdh5-Cre*^*ERT2*^ mice, and *Myh9*^*iECKI*^ mice were generated via CRISPR/Cas9 system. Tamoxifen (150 mg/kg) was administered intraperitoneally for 5 consecutive days to induce endothelial cell-specific *Myh9*^*ECKD*^ mice. Genotype of mice was analyzed 2 weeks after tamoxifen injection by PCR assay. Genotyping primers for *Myh9*^*ECKD*^ and *Myh9*^*iECKI*^ mice were provided in Supplementary Material (Supplementary Table 1). The mice were housed for 3–5 days in SPF-grade independent ventilated cages (IVC) to acclimate to the environment, with unrestricted access to food and water. The room conditions were maintained at a temperature of 23–25 °C, humidity between 40% and 80%, and a 12-hour light-dark cycle.

Male wild-type C57BL/6J mice (SPF grade, 3–8 weeks old, 18–22 g) were obtained from Jiangsu Huachuang Xinnuo Pharmaceutical Technology Co., Ltd. (certificate number: SYXK 2020-0009; Nanjing, China) and housed under the same conditions as the *Myh9*^*ECKD*^ mice. All experimental protocols were conducted in compliance with National Institutes of Health guidelines and were approved by the Animal Ethics Committee of China Pharmaceutical University (Nanjing, China, No.2023-03-023). Mice were randomly grouped before experimentation using a simple randomization method.

### MCAO/R model

The MCAO/R model was prepared as described previously [11]. Briefly, mice were anesthetized in an induction chamber with 2% isoflurane in a 30% O_2_ and 70% N_2_ gas mixture. Anesthesia was maintained with 1-1.5% isoflurane via a nose mask. Body temperature was controlled at 37 ± 0.5 °C during the procedure. Following a midline cervical incision, the right common carotid artery (rCCA), external carotid artery (rECA), and internal carotid artery (rICA) were isolated. To occlude the blood supply to the right cerebral hemisphere, a commercial 0622 nylon monofilament was inserted into the rICA until the tip reached 1–2 mm from the origin of the right middle cerebral artery (rMCA). After 1 h of occlusion, the filament was withdrawn to allow reperfusion, and the rCCA was ligated. Sham group mice underwent the same procedure without occlusion. Laser Doppler flowmetry (LDF; FLPI2, Moor, UK) was used to verify model success, with mice excluded if rMCA blood flow did not decrease below 30% of baseline after occlusion [18]. For drug administration, blebbistatin (10 mg/kg) or Y-27632 (10 mg/kg) was administered intraperitoneally after 1 h of reperfusion. After 24 h of reperfusion, all mice were assessed and euthanized.

### Cell culture

Primary brain microvascular endothelial cells (pBMECs) were isolated as previously described [19]. Brain-derived endothelial cells (bEnd.3) were provided by the Shanghai Institute of Cell Biology, Chinese Academy of Science, and cultured in Dulbecco’s Modified Eagle Medium (DMEM; Gibco) supplemented with 10% fetal bovine serum (FBS; ExCell), 100 U/mL penicillin, and 100 U/mL streptomycin. Cells were maintained at 37 °C in a humidified incubator with 5% CO_2_ and 95% air, and were allowed to reach 80–90% confluence before experiments.

### Oxygen and glucose deprivation/reperfusion model

To mimic cerebral I/R in vitro, bEnd.3 and pBMECs were subjected to OGD/R. Cells were cultured in glucose-free medium and incubated in a 37 °C anaerobic chamber, continuously supplied with 1% O_2_, 5% CO_2_, and 94% N_2_, for 6 h. For reperfusion, cells were transferred to glucose-containing DMEM and incubated in a normoxic atmosphere (5% CO_2_, 95% air) for 18 h. The control group was cultured in normal medium under normoxic conditions for 24 h. Drug treatment involved diluting blebbistatin (1 µM), Y-27632 (1 µM), and cytochalasin D (0.1 µM) in culture medium after OGD.

### TTC staining

To assess infarct size, 2, 3, 5-triphenyl tetrazolium chloride (TTC) staining was performed. After 1 h of ischemia and 24 h of reperfusion, mice were euthanized, and their brains were excised. The brains were frozen at −20 °C for 2 h, sectioned into 2-mm-thick slices using a rodent brain matrix, and stained with 1.2% TTC in PBS at 37 °C for 30 min, protected from light. The infarcted areas were photographed and analyzed using ImageJ software. The infarct ratio was calculated as the ipsilateral infarct area relative to the total contralateral area.

### Neurological deficit evaluation

Neurological deficits were evaluated 24 h after reperfusion using a five-point scale as previously described [20]: 0 = no deficits; 1 = failure to fully extend the contralateral forepaw; 2 = circling behavior toward the ipsilateral side; 3 = falling to the contralateral side; 4 = no movement or minimal consciousness. Higher scores indicate more severe neurological impairment.

### Evans blue (EB) permeability assay

BBB permeability was assessed using EB extravasation, as previously described [20]. After 22 h of reperfusion, 2% EB solution (Sigma Aldrich) in saline was injected via the tail vein (0.1 mL/10 g body weight). After 2 h of circulation, mice were euthanized and perfused with PBS. The brains were harvested, photographed, and the ischemic hemisphere was homogenized in formamide (1 mL: 0.1 g). The samples were incubated at 60 °C for 18 h, centrifuged at 5, 000 g for 30 min, and the supernatant’s absorbance at 620 nm was measured. EB content was calculated using a standard curve (µg/g tissue).

### Western blot analysis

Total protein from ischemic penumbra brain tissues, pBMECs, and bEnd.3 cells (*n* = 5 per group) were extracted using RIPA buffer supplemented with 1% PMSF and 2% phosphatase inhibitors. Protein concentrations were quantified by BCA assay. Equal amounts of protein (30 µg) were separated by 10% polyacrylamide gel electrophoresis (PAGE) and transferred onto polyvinylidene fluoride (PVDF) membranes. After blocking with 5% BSA in TBST for 2 h, membranes were incubated overnight at 4 °C with primary antibodies (ROCK1, NMMHC IIA: 1:8000, Proteintech; p-MLC2, MLC2: 1:1000, CST; ZO-1: 1:2000, Proteintech; Occludin: 1:10000, Proteintech; F-actin: 1:3000, Abcam; GAPDH: 1:10000, Bioworld). Secondary antibodies (goat anti-rabbit, goat anti-mouse: 1:10000, Bioworld) were applied for 1.5 h at room temperature, and proteins were visualized using ECL and analyzed with Image Lab software.

### Immunofluorescence

Frozen ischemic penumbra brain sections (10 μm) and cultured pBMECs or bEnd.3 cells were fixed in 4% paraformaldehyde for 15 min, permeabilized with Triton X-100 for 10 min, and blocked with commercial blocking buffer at 25 °C for 30 min. Samples were incubated overnight at 4 °C with primary antibodies against ROCK1 (1:200, Proteintech), NMMHC IIA (1:200, Proteintech), F-actin (1:100, Abcam), and CD31 (1:200, R&D Systems). Secondary antibodies (1:200, Invitrogen) or Alexa Fluor^®^ 568 phalloidin (1:100, Thermo Fisher) were applied at 25 °C for 2 h, followed by DAPI staining. Fluorescent images were captured using a laser confocal microscope (LSM700; Zeiss) and analyzed with ZEN software. To quantify co-localization of NMMHC IIA and F-actin, images were analyzed using ImageJ software. Background noise was subtracted, and Manders’ overlap coefficients were calculated using the JACoP plugin, with values ranging from 0 (no correlation) to 1 (complete overlap).

### Co-immunoprecipitation (Co-IP)

The Co-IP experiments were conducted as previously described [16]. In summary, 50 µL of Protein A/G PLUS-Agarose was washed 4–5 times with RIPA buffer, centrifuged at 3000 rpm for 3 min, and the supernatant was discarded. To facilitate antibody coupling, 2 µg of antibody was added to the agarose suspension and incubated overnight at 4 °C with continuous rotation. The antibody-agarose complex was subsequently mixed with 1 mL of whole cell lysate (1 µg/µL) and incubated for 6 h at 4 °C on a rotator. After four washes with RIPA buffer, the samples were mixed with 2 × loading buffer and boiled at 100 °C for 5 min. The proteins were then analyzed by Western blot.

### Statistical analysis

All experimental data are presented as mean ± standard deviation (SD). Statistical comparisons between groups were performed using GraphPad Prism 8. One-way analysis of variance (ANOVA), followed by Dunnett’s post-hoc test, was employed to assess significance. *P* value of less than 0.05 was considered statistically significant.

## Results

### *Myh9*^*ECKD*^ suppresses ROCK/MLC/NMMHC IIA-actin activation induced by MCAO/R in mice

Previous studies have demonstrated that *Myh9*^*ECKD*^ attenuates MCAO/R-induced brain injury and preserves BBB integrity [11]. To evaluate the neuroprotective and BBB-preserving effects of NMMHC IIA inhibition, we subjected *Myh9*^*ECKD*^ mice to MCAO/R. 24 h post-reperfusion, neurological deficit scores were recorded, and the mice were sacrificed for the assessment of cerebral infarct volume, EB extravasation, TJs and Claudin-5 expression. *Myh9*^*ECKD*^ significantly improved neurological function and reduced infarct size compared to the I/R group (Supplementary Fig. S1a-c). Moreover, the *Myh9*^*ECKD*^ group exhibited significantly reduced EB leakage and preserved ZO-1, occludin and Claudin-5 protein levels, indicative of attenuated BBB disruption (Supplementary Fig. S1d-g, S8a). To further elucidate the role of NMMHC IIA, we used blebbistatin, a selective non-muscle myosin II inhibitor, to assess its effect on BBB integrity and ischemic stroke in vivo. Likewise, C57BL/6J mice given blebbistatin 1 h after ischemia also showed significant improvements in cerebral infarct volume, neurological function, EB leakage, TJs degradation and fluorescein isothiocyanate (FITC)-dextran (40 kD) leakage (Supplementary Fig. S2a-g, S6a-b).

**Fig. 1:**
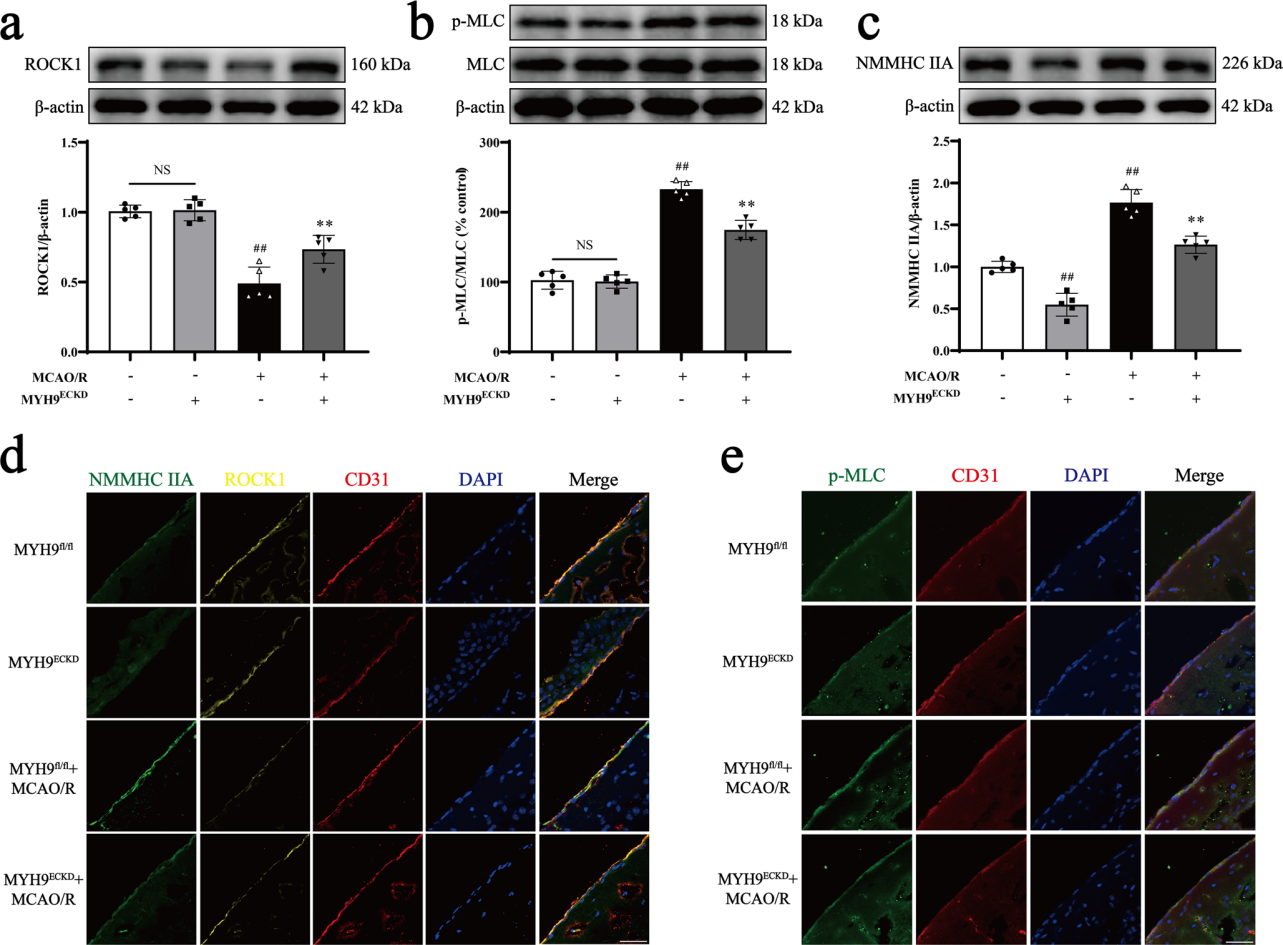
*Myh9*^*ECKD*^ inhibits MCAO/R-induced activation of the ROCK/MLC/NMMHC IIA-actin pathway in mice. Mice received tamoxifen (150 mg/kg) for 5 consecutive days, followed by 1 h of ischemia and 24 h of reperfusion. **a**,** b**,** c** Representative Western blot images and quantitative analyses of ROCK1, phosphorylated MLC (p-MLC), total MLC, and NMMHC IIA expression. **d** Immunofluorescence images showing NMMHC IIA (green), ROCK1 (yellow), and CD31 (red), with nuclei stained by DAPI (blue). **e** Immunofluorescence images of p-MLC (green) and CD31 (red), with nuclei stained by DAPI (blue). Data are presented as mean ± SD, *n* = 5. Statistical significance is denoted by NS (non-significant), ^##^*P* < 0.01 vs. *Myh9*^*fl/fl*^ group, ^**^P < 0.01 vs. *Myh9*^*fl/fl*^ + MCAO/R group. Scale bar, 50 μm

It has been reported that the ROCK/MLC pathway is essential for cytoskeletal reorganization and apoptosis [21], and that NMMHC IIA inhibition can mitigate H_2_O_2_-induced Caspase-3/ROCK/MLC pathway activation [16]. Therefore, we examined the effect of *Myh9*^*ECKD*^ and blebbistatin on the cytoskeleton-related ROCK1/MLC pathway. Western blot analysis revealed that I/R significantly decreased ROCK1 expression and increased MLC phosphorylation compared to *Myh9*^*fl/fl*^ mice. However, *Myh9*^*ECKD*^ and blebbistatin significantly restored ROCK1 expression and suppressed MLC phosphorylation relative to the I/R group (Fig. 1a-c; Supplementary Fig. S2h-j). Immunofluorescence analysis produced consistent results (Fig. 1d-e).

### *Myh9*^*ECKD*^ inhibit ROCK/MLC/NMMHC IIA-actin activation induced by OGD/R in pBMECs

**Fig. 2:**
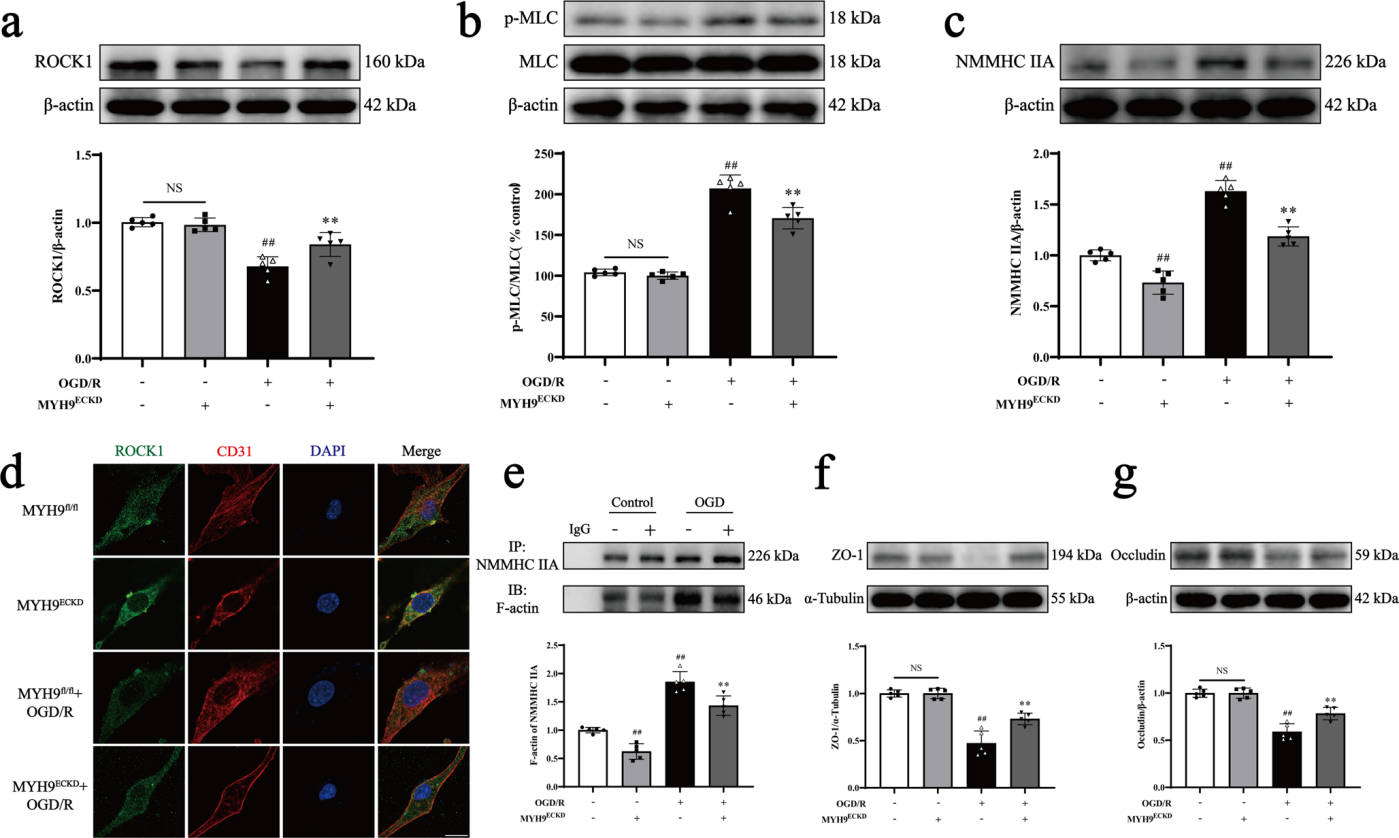
Effects of *Myh9*^*ECKD*^ on the activation of ROCK/MLC/NMMHC IIA-actin pathway in pBMECs subjected to OGD/R. *Myh9*^*ECKD*^ pBMECs were exposed to 6 h of OGD followed by 18 h of reoxygenation. **a**,** b**,** c** Western blot analysis of ROCK1, p-MLC, MLC, and NMMHC IIA expression. **d** Immunofluorescence images of ROCK1 (green) and CD31 (red), with nuclei stained by DAPI (blue). **e** Co-IP analysis of NMMHC IIA and F-actin interaction. **f**,** g** Western blot analysis and quantitative assessment of ZO-1 and occludin levels in *Myh9*^*fl/fl*^ and *Myh9*^*ECKD*^ pBMECs. Data are presented as mean ± SD, *n* = 5. Statistical significance is denoted by NS (non-significant), ^##^*P* < 0.01 vs. *Myh9*^*fl/fl*^ group, ^**^P < 0.01 vs. *Myh9*^*fl/fl*^ + OGD/R group. Scale bar, 50 μm

Next, we evaluated the effects of *Myh9*^*ECKD*^ on the ROCK/MLC/NMMHC IIA-actin axis in pBMECs exposed to OGD/R. Western blot analysis demonstrated that OGD/R significantly increased MLC phosphorylation and reduced ROCK1 expression compared to controls. *Myh9*^*ECKD*^ reversed these changes, inhibiting both MLC phosphorylation and the downregulation of ROCK1 in pBMECs (Fig. 2a-c). Immunofluorescence analysis further corroborated the increased ROCK1 expression in *Myh9*^*ECKD*^ cells (Fig. 2d). The interaction between NMMHC IIA and F-actin is required to initiate actomyosin contractility, a key mechanism in BBB disruption. Co-IP assays revealed that OGD/R increased the interaction between NMMHC IIA and F-actin, while *Myh9*^*ECKD*^ inhibited this interaction (Fig. 2e). Pretreatment with blebbistatin (1 µM) for 6 h prior to OGD/R in bEnd.3 cells similarly inhibited activation of the ROCK/MLC signaling pathway and the interaction between NNMHC IIA and F-actin (Supplementary Fig. S3a-e, S7). Additionally, *Myh9*^*ECKD*^ and blebbistatin attenuated the degradation of the TJs of ZO-1 and occludin in pBMECs and bEnd.3 cells following OGD/R, contributing to BBB protection (Fig. 2f-g; Supplementary Fig. S3f-g).

### *Myh9*^*iECKD*^ exacerbates BBB leakage after ischemic stroke in mice

**Fig. 3:**
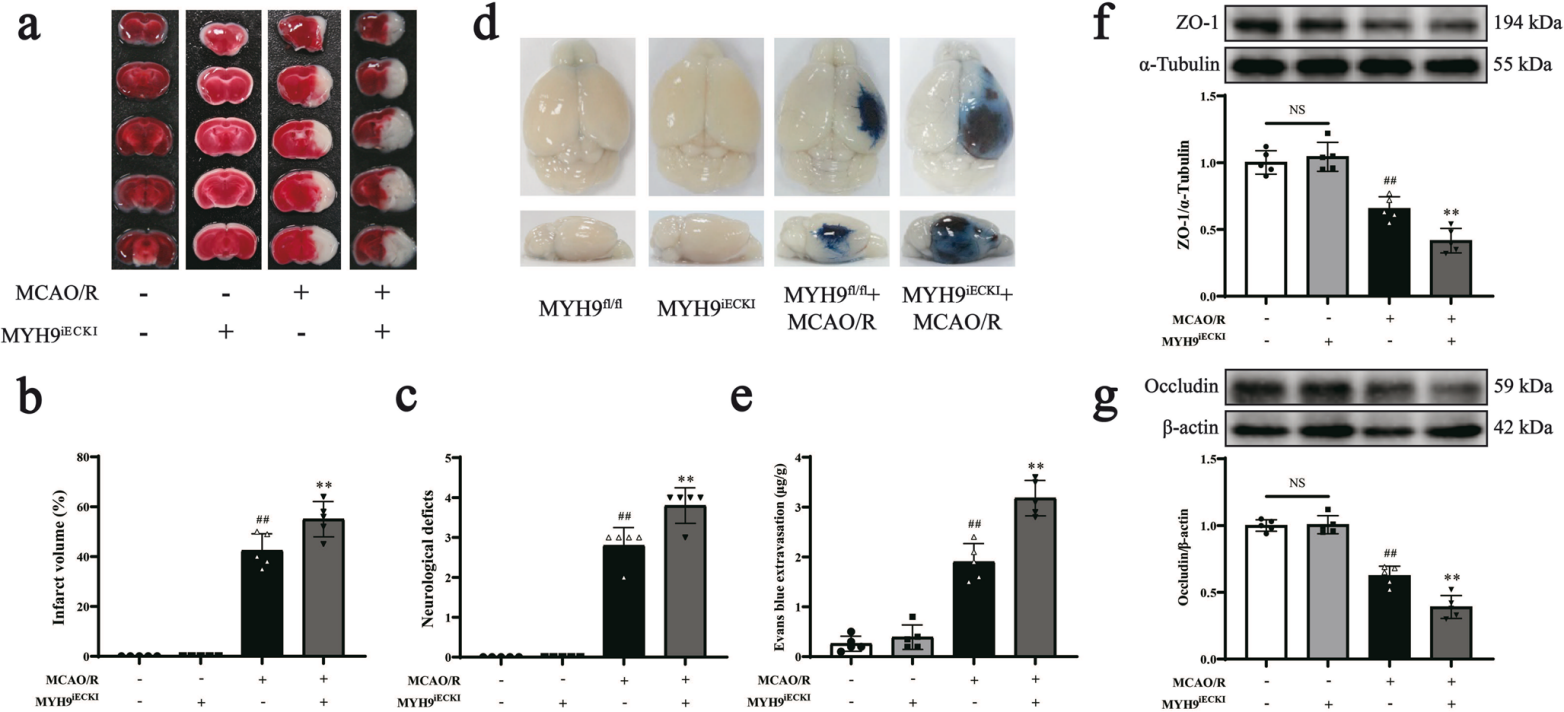
*Myh9*^*iECKI*^ exacerbates BBB leakage after ischemic stroke in mice. Mice were subjected to 1 h of ischemia and 24 h of reperfusion. **a** Representative brain sections stained with TTC, where infarcted areas appear white and non-infarcted regions red. **b** Quantitative analysis of infarct volume was performed for all experimental groups. **c** Representative neurological deficit scores, where higher scores correspond to more severe impairments. **d**,** e** Representative gross images of EB-stained brains, along with a quantitative assessment of EB extravasation (µg). **f**,** g** Representative Western blot data and quantitative analysis of the expression levels of the TJs of ZO-1 and occludin. Data are presented as mean ± SD, *n* = 5. Statistical significance is denoted by NS (non-significant), ^##^*P* < 0.01 vs. *Myh9*^*fl/fl*^ group, ^**^P < 0.01 vs. *Myh9*^*fl/fl*^ + MCAO/R group

Above results have shown that inhibition of NMMHC IIA expression in endothelial cells significantly ameliorated MCAO/R-induced ischemia brain damage and BBB destruction. To test the effects of overexpression of NMMHC IIA on I/R-induced BBB leakage, we constructed *Myh9*^*iECKI*^ mice. The overexpression efficiency of NMMHC IIA was about 150–160% in *Myh9*^*iECKI*^ mice according to PCR and western blot experiments (Supplementary Fig. S4a-b). The results revealed that *Myh9*^*iECKI*^ further deteriorated cerebral infarct size and neurological deficits after MCAO/R (Fig. 3a-c). We further evaluated BBB leakage by intravenously (i.v.) injecting EB into stroke mice after reperfusion 22 h. The leakage of EB was higher in *Myh9*^*iECKI*^ compared to *Myh9*^*fl/fl*^ mice after MCAO/R (Fig. 3d, e). TJs degradation is an important characterization in the pathological process of BBB damage, therefore, it was used as an index to evaluate the leakage of BBB. Compared with the MCAO/R group, *Myh9*^*iECKI*^ aggravated the degradation of TJs of ZO-1 and occludin (Fig. 3f-g).

### *Myh9*^*iECKI*^ aggravates ROCK/MLC/NMMHC IIA-actin activation induced by MCAO/R in mice

**Fig. 4:**
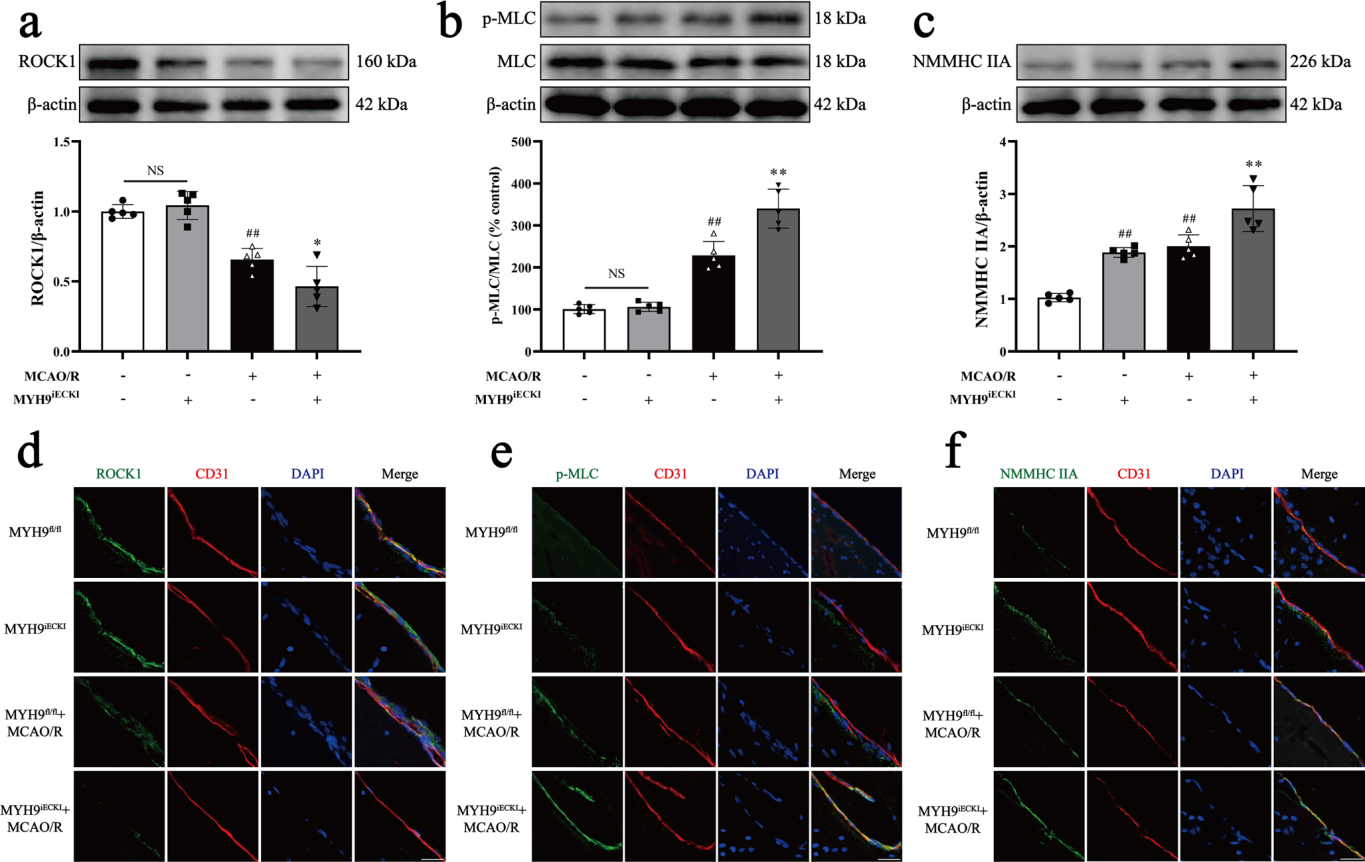
*Myh9*^*iECKI*^ aggravates MCAO/R-induced activation of the ROCK/MLC/NMMHC IIA-actin pathway. Mice were subjected to 1 h of ischemia and 24 h of reperfusion. **a**,** b**,** c** Western blot images and quantitative analyses of ROCK1, p-MLC, total MLC, and NMMHC IIA expression. **d** Immunofluorescence images showing ROCK1 (green) and CD31 (red), with nuclei stained by DAPI (blue). **e** Immunofluorescence images of p-MLC (green) and CD31 (red), with nuclei stained by DAPI (blue). **f** Immunofluorescence images of NMMHC IIA (green) and CD31 (red), with nuclei stained by DAPI (blue). Data are presented as mean ± SD, *n* = 5. Statistical significance is denoted by NS (non-significant), ^##^*P* < 0.01 vs. *Myh9*^*fl/fl*^ group, ^*^P < 0.05, ^**^P < 0.01 vs. *Myh9*^*fl/fl*^ + MCAO/R group. Scale bar, 50 μm

We then explored the activation of ROCK/MLC/NMMHC IIA signaling pathway in *Myh9*^*iECKI*^ mice after MCAO/R. Western blot analysis revealed that I/R significantly decreased ROCK1 expression and increased NMMHC IIA expression and MLC phosphorylation compared to *Myh9*^*fl/fl*^ mice. *Myh9*^*iECKI*^ did not activate the ROCK/MLC pathway under normal conditions, however, *Myh9*^*iECKI*^ further inhibited ROCK1 expression and increased NMMHC IIA expression and MLC phosphorylation relative after MCAO/R (Fig. 4a-c). Immunofluorescence analysis produced consistent results (Fig. 4d-f). Taken together, overexpression of NMMHC IIA further amplified the activation of the ROCK/MLC signaling pathway, suggesting the possibility that NMMHC IIA is involved in cerebral I/R-induced BBB injury through a positive feedback regulatory loop.

### The ROCK1 inhibitor Y-27632 ameliorate BBB leakage after ischemic stroke in mice

**Fig. 5:**
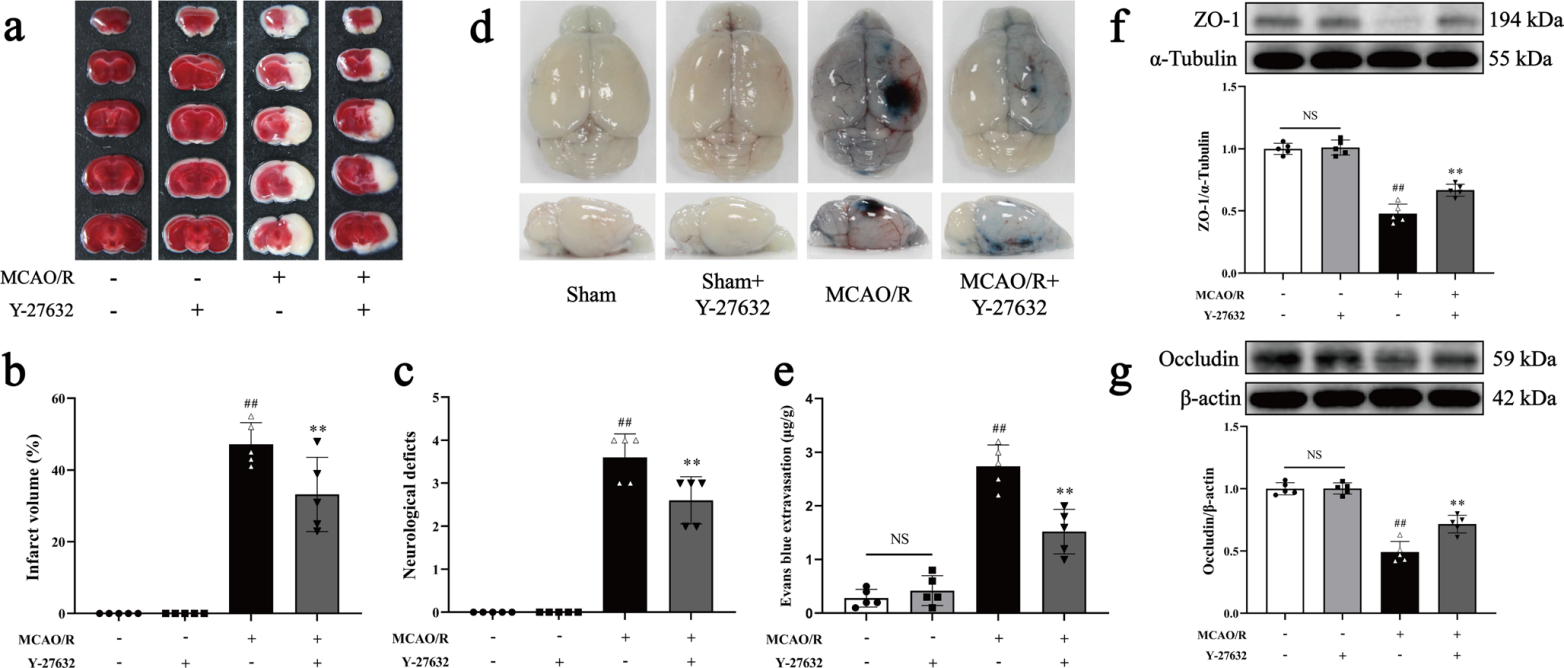
Effects of Y-27632 on MCAO/R-induced ischemic stroke in mice. Mice were subjected to 1 h of ischemia followed by 24 h of reperfusion. Y-27632 (10 mg/kg, i.p.) was administered 1 h post-ischemia. **a** Representative brain sections stained with TTC, where infarcted areas appear white and non-infarcted regions red. **b** Quantitative analysis of infarct volume was performed for all experimental groups. **c** Representative neurological deficit scores, where higher scores correspond to more severe impairments. **d**,** e** Representative gross images of EB-stained brains, along with a quantitative assessment of EB extravasation (µg). **f**,** g** Representative Western blot data and quantitative analysis of the expression levels of the TJs of ZO-1 and occludin. Data are expressed as mean ± SD, with *n* = 5. Statistical significance is denoted by NS (non-significant), ^##^*P* < 0.01 compared to the Sham group, and ^**^P < 0.01 compared to the MCAO/R group

The activation of the ROCK/MLC signaling cascade is well-documented in ischemic stroke, where ROCK promotes stress fiber formation and cytoskeletal reorganization by phosphorylating MLC, thereby enhancing the interaction between NMMHC IIA and F-actin, a key cytoskeletal component [22]. To investigate the involvement of this signaling loop in stroke pathology, the ROCK1 inhibitor Y-27632 was employed. However, whether Y-27632 improves cerebral I/R injury is unclear. In vivo experiments demonstrated that administering Y-27632 at a dose of 10 mg/kg following ischemia significantly reduced cerebral infarct volume and improved neurological outcomes in MCAO/R mice (Fig. 5a-c). Additionally, Y-27632 substantially mitigated BBB disruption by transcellular transport and paracellular permeability, as evidenced by reduced EB leakage, FITC-dextran (40 kD) leakage, and preserved expression of ZO-1, occludin, and Claudin-5 (Fig. 5d-g; Supplementary Fig. S6a-b, S8b). Similarly, Y-27632 also preserved tight junction integrity by reducing the degradation of ZO-1 and occludin in bEnd.3 cells exposed to OGD/R (Supplementary Fig. S5g-h). Taken together, the results suggest that Y-27632 ameliorates BBB leakage after I/R.

### Y-27632 suppresses MCAO/R-induced activation of the ROCK/MLC/NMMHC IIA-actin pathway

**Fig. 6:**
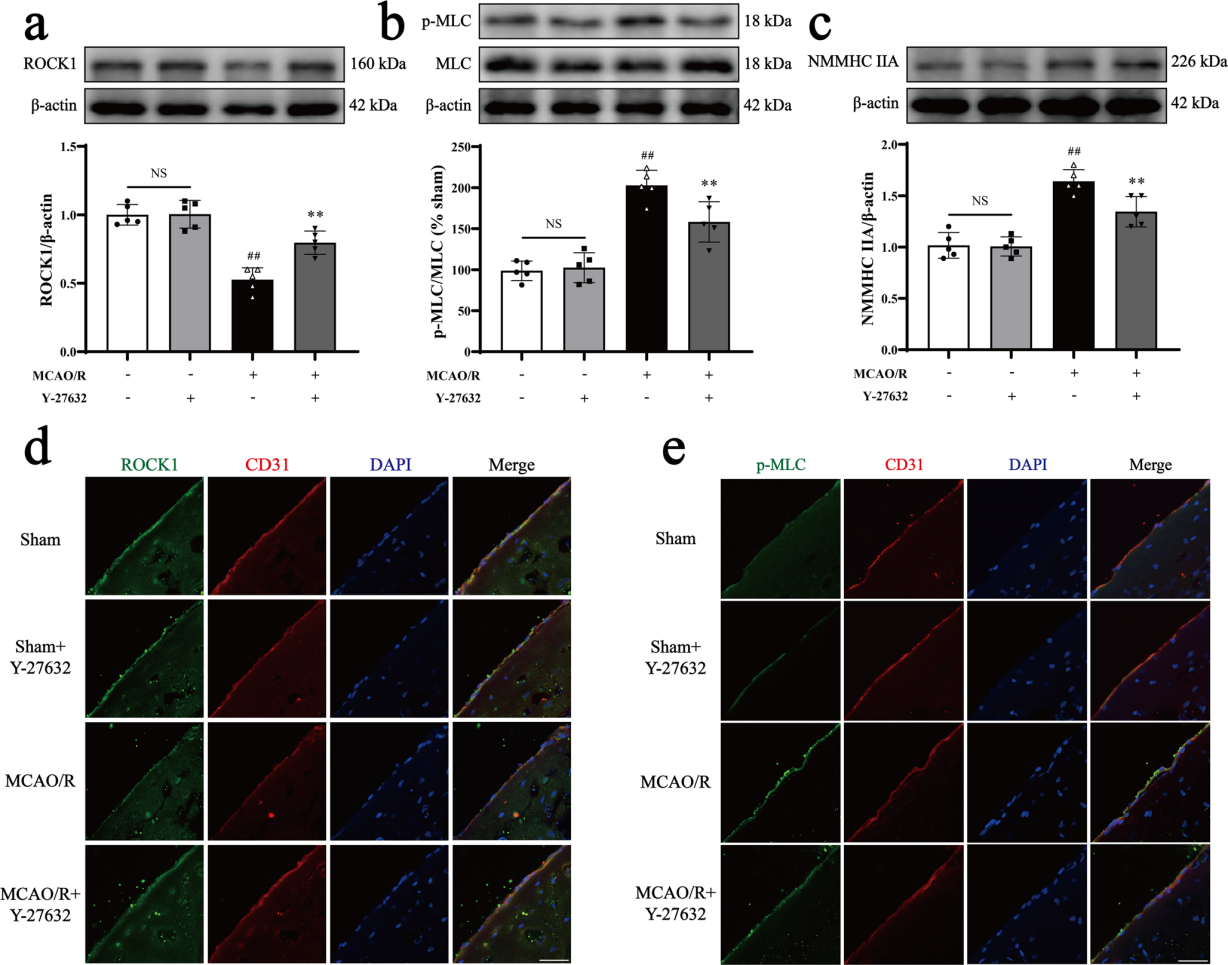
The ROCK1 inhibitor Y-27632 suppresses MCAO/R-induced activation of the ROCK/MLC/NMMHC IIA-actin pathway in mice. Mice were subjected to 1 h of ischemia followed by 24 h of reperfusion. Y-27632 (10 mg/kg, i.p.) was administered 1 h post-ischemia. **a**,** b**,** c** Western blot results and quantitative analyses of ROCK1, p-MLC, MLC, and NMMHC IIA. **d** Representative immunofluorescence images of ROCK1 (green) and CD31 (red), with nuclei stained by DAPI in blue. **e** Representative immunofluorescence images of p-MLC (green) and CD31 (red). Data are expressed as mean ± SD, with *n* = 5. Statistical significance is denoted by NS (non-significant), ^##^*P* < 0.01 compared to the Sham group, and ^**^P < 0.01 compared to the MCAO/R group. Scale bar, 50 μm

To elucidate the molecular mechanisms underlying this effect, we examined the expression of phosphorylated MLC and NMMHC IIA after administration of Y-27632 following MCAO/R. Western blot analysis confirmed that Y-27632 inhibited the MCAO/R-induced upregulation of NMMHC IIA and MLC phosphorylation while preventing the downregulation of ROCK1 (Fig. 6a-c). Immunofluorescence analysis further corroborated that administration of Y-27632 decreased ROCK1 and p-MLC expression in MCAO/R mice (Fig. 6d-e).

To further assess the role of Y-27632 at the cellular level, we investigated its impact on bEnd.3 cells subjected to OGD/R. Under normoxic conditions, Y-27632 did not significantly alter ROCK1 expression and activity. However, in OGD/R conditions, Y-27632 markedly restored ROCK1 expression and suppressed ROCK activity (Supplementary Fig. S5a, d, S7). Western blot analysis revealed that Y-27632 attenuated both NMMHC IIA expression and MLC phosphorylation in OGD/R-treated cells (Supplementary Fig. S5b-c). Furthermore, the increased interaction between NMMHC IIA and F-actin observed after stroke was significantly inhibited following Y-27632 administration, as demonstrated by immunofluorescence and Co-IP (Supplementary Fig. S5e-f). Collectively, these findings validate the neuroprotective efficacy of Y-27632 through inhibition of the ROCK1/MLC/NMMHC IIA signaling axis in ischemic stroke.

### Cytochalasin D inhibits ROCK/MLC/NMMHC IIA-actin activation induced by OGD/R in bEnd.3 cells

**Fig. 7:**
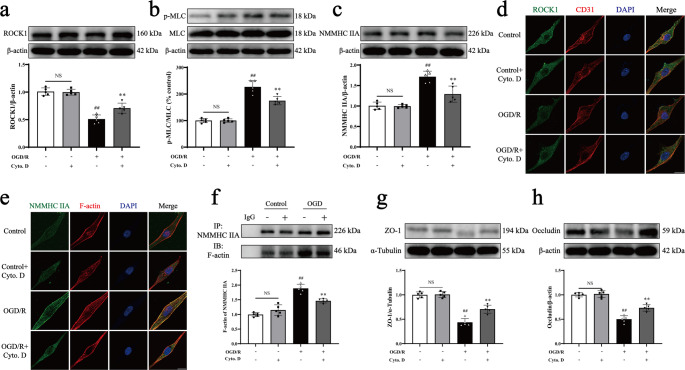
Protective effects of cytochalasin D on OGD/R-induced ROCK/MLC/NMMHC IIA-actin activation in bEnd.3 cells. bEnd.3 cells were subjected to OGD/R and treated with cytochalasin D (0.1 µM) for 18 h after OGD. **a**,** b**,** c** Representative Western blot data and quantitative analyses of ROCK1, p-MLC, MLC, and NMMHC IIA expression. **d** Representative immunofluorescence images of ROCK1 (green) and CD31 (red), with DAPI-stained nuclei in blue. **e** Representative immunofluorescence images of NMMHC IIA (green) and F-actin (red), with nuclei stained by DAPI in blue. **f** Co-IP results for the interaction between NMMHC IIA and F-actin under OGD/R conditions. **g**,** h** Representative Western blot data and quantitative analyses of ZO-1 and occludin expression. Data are represented as mean ± SD, *n* = 5. Statistical significance is denoted by NS (non-significant), ^##^*P* < 0.01 compared to the Control group, and ^**^P < 0.01 compared to the OGD/R group. Scale bar, 50 μm

Previous studies have shown that NMMHC IIA and F-actin form stress fibers, which facilitate cytoskeletal rearrangement and exacerbate I/R injury. Cytochalasin D, an actin depolymerization agent, disrupts these stress fibers by binding to actin [23]. To confirm the role of the NMMHC IIA-actin loop in stroke, cytochalasin D was employed. The results indicated that cytochalasin D significantly reduced the expression of NMMHC IIA, inhibited MLC phosphorylation, suppressed ROCK1 expression and activity in OGD/R-treated bEnd.3 cells (Fig. 7a-d, S7). Immunofluorescence and Co-IP analyses revealed that the interaction between NMMHC IIA and F-actin, which was elevated after stroke, was notably diminished following cytochalasin D treatment (Fig. 7e-f). Additionally, cytochalasin D effectively prevented the degradation of ZO-1 and occludin, thereby preserving BBB integrity in OGD/R-treated cells (Fig. 7g-h).

**Fig. 8:**
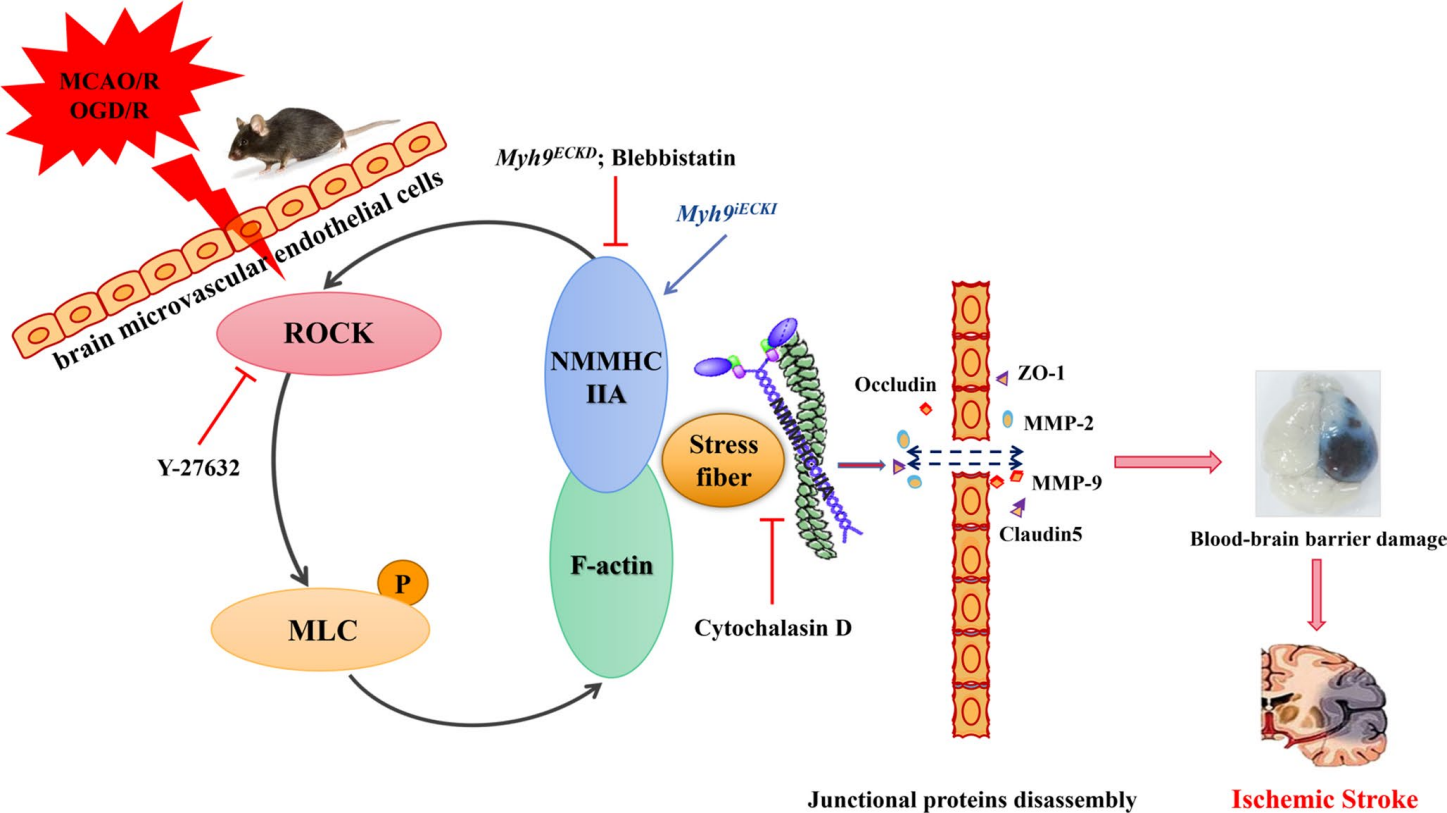
Inhibition of the ROCK1/MLC/NMMHC IIA-actin feedback loop attenuates BBB disruption and cerebral I/R injury

## Disscusion

In this study, we demonstrated that the ROCK/MLC/NMMHC IIA-actin signaling loop plays a pivotal role in cerebral I/R injury by exacerbating BBB dysfunction. Following I/R, activation of the ROCK/MLC pathway enhances the actin-myosin interaction, resulting in the formation of stress fibers that stretch the cytoskeleton, destabilize tight junctions between endothelial cells, and increase BBB permeability [24]. Notably, the ROCK inhibitor Y-27632 attenuated these effects by suppressed ROCK activity, reducing MLC phosphorylation, NMMHC IIA expression, and the interaction between NMMHC IIA and F-actin, thereby mitigating I/R-induced BBB damage (Figs. 5 and 6; Supplementary Fig. S5, S7).

The positive feedback loop between ROCK/MLC and NMMHC IIA-actin was further explored through inducible endothelial conditional knock-in and endothelial-specific knockdown of NMMHC IIA, as well as through the use of the NMMHC II inhibitor blebbistatin and the F-actin depolymerizer cytochalasin D. Both the conditional knockdown of NMMHC IIA and treatment with blebbistatin effectively suppressed the ROCK/MLC signaling pathway, reduced the NMMHC IIA and F-actin interaction, and improved BBB integrity following I/R (Figs. 1 and 2; Supplementary Fig. S1, S2, S3, S6, S7, S8). Conversely, endothelial conditional knock-in of NMMHC IIA aggravated the activation of ROCK/MLC signaling pathway, and accentuated BBB disruption following MCAO/R (Figs. 3 and 4). Additionally, cytochalasin D inhibited the interaction between NMMHC IIA and F-actin, downregulated NMMHC IIA expression, suppressed the ROCK/MLC signaling pathway, and prevented the degradation of TJs in bEnd.3 cells subjected to OGD/R (Fig. 7, Supplementary Fig. S7). Collectively, these findings indicate that the ROCK/MLC/NMMHC IIA-actin loop contributes significantly to ischemic stroke and BBB disruption, and its inhibition represents a novel therapeutic strategy for IS treatment.

NMMHC IIA, an isoform of non-muscle myosin II encoded by the *MYH9* gene [25], functions as a key molecular motor and cytoskeletal protein. It plays a crucial role in various physiological and pathological processes, including cell adhesion, migration, tumor metastasis, thrombosis, and apoptosis, and is implicated in several diseases such as chronic kidney disease, deafness, and cancer [26–28]. Previous studies have shown that NMMHC IIA modulates tissue factor and the Akt/GSK3β-NF-κB signaling pathways in the endothelium to prevent venous thrombosis [28–31], suggesting its potential as a therapeutic target for inflammation-related cardiovascular diseases. NMMHC IIA expression is upregulated in endothelial cells following I/R, and endothelial-specific knockdown of NMMHC IIA or administration of blebbistatin improves BBB hyperpermeability induced by MCAO/R (Supplementary Fig. S1, S2, S6, S8) [11, 32]. Conversely, *Myh9*^*iECKI*^ further exacerbate cerebral I/R injury and BBB damage (Fig. 3). Furthermore, transcriptomic analyses have identified NMMHC IIA as a regulator of numerous genomic changes related to BBB integrity following I/R [11]. These data suggest a close association between NMMHC IIA and BBB injury post-I/R.

Mounting evidence indicates that BBB disruption is closely linked to endothelial cytoskeletal rearrangement following cerebral I/R [8, 33]. Multiple signaling pathways are involved in regulating cytoskeletal remodeling [34, 35], among which the RhoA/ROCK pathway serves as a critical molecular switch for controlling cytoskeletal dynamics [36, 37]. ROCK1, a well-characterized effector of RhoA, promotes stress fiber formation and exacerbates intercellular gap formation by phosphorylating MLC and enhancing the interaction between F-actin and NMMHC IIA [38, 39]. In this study, we observed a significant downregulation of ROCK1 expression and an upregulation of p-MLC and NMMHC IIA expression, accompanied by an increased interaction between NMMHC IIA and F-actin in endothelial cells following I/R. Importantly, treatment with Y-27632 markedly inhibited ROCK/MLC pathway activation and the formation of NMMHC IIA-actin-mediated stress fibers (Fig. 6a-e; Supplementary Fig. S5a-f). These findings align with previous studies [38, 39], which have reported that pharmacological inhibition of ROCK1 with Y-27632 alleviates the deleterious effects of TNF-α on BBB dysfunction in vitro [40]. Similarly, we found that Y-27632 reduced EB and FITC-dextran (40 kDa) leakage, prevented the degradation of ZO-1, occludin and Claudin-5 following I/R by influencing both transcellular transport and paracellular permeability (Fig. 5d-g; Supplementary Fig. S5g-h, S6a-b, S8b). Additionally, Y-27632 improved cerebral infarct volume and neurological outcomes post-MCAO/R (Fig. 5a-c). Collectively, these results suggest that the ROCK/MLC signaling pathway regulates NMMHC IIA-actin-mediated stress fiber formation, contributing to BBB disruption and I/R injury.

Previous studies have also demonstrated that the NMMHC IIA-actin interaction mediates I/R-induced neuronal apoptosis and activates the Caspase-3/ROCK1/MLC pathway in a positive feedback loop, thereby amplifying actomyosin contractility [16]. However, the role of the NMMHC IIA-actin interaction in regulating the ROCK/MLC pathway through positive feedback in endothelial cell-mediated BBB injury following I/R remains unclear. To investigate this, we utilized blebbistatin, cytochalasin D and *Myh9*^*ECKD*^ mice. Our results revealed that inhibition or knockdown of NMMHC IIA in endothelial cells significantly suppressed the ROCK/MLC pathway (Figs. 1a-e and 2a-e; Supplementary Fig. S2h-j, S3a-e, S7). Cytochalasin D, a fungal metabolite that specifically binds actin and disrupts its interaction with myosin, effectively reduced stress fiber formation [41]. This treatment inhibited NMMHC IIA-actin interaction, prevented the degradation of TJs, and blocked matrix metalloproteinase (MMP) activity, ultimately preserving BBB integrity and reducing I/R injury [21, 42]. We demonstrated that cytochalasin D protected the BBB by inhibiting the degradation of ZO-1 and occludin (Fig. 7g-h), as well as reducing the interaction between NMMHC IIA and actin, and suppressing the activation of the ROCK/MLC/NMMHC IIA pathway (Fig. 7a-f, S7). These findings provide the first evidence that the ROCK/MLC/NMMHC IIA-actin positive feedback loop is implicated in I/R-induced BBB disruption (Fig. 8). Targeting the ROCK/MLC/NMMHC IIA-actin signaling pathway presents a promising strategy for treating ischemic stroke and BBB-related diseases, including cerebral hemorrhage, Parkinson’s disease, and Alzheimer’s disease, etc. However, it remains unclear whether endothelial NMMHC IIA knockdown in other tissues exacerbates or mitigates brain I/R injury, warranting further investigation through multi-organ transcriptomics, parabiosis experiments, and tissue-specific knockout models to elucidate potential multi-organ endothelial crosstalk. Additionally, while this study investigantes the regulatory role of endothelial cells predominantly expressing NMMHC IIA in cerebral IR-induced BBB disruption via the loop mechanism, the effects of BBB-related cells, including astrocytes, pericytes and basement membrane, on this process remains unclear. Future studies employing advanced tri-culture models and brain organoid systems could help elucidate the multicellular regulatory mechanisms governing NMMHC IIA function at the BBB.

## Conclusion

In conclusion, this study reveals that the ROCK/MLC/NMMHC IIA-actin positive feedback loop is crucial in BBB disruption following cerebral I/R injury. Targeting this signaling axis may offer a novel therapeutic approach to protecting the BBB and treating ischemic stroke.

## Electronic supplementary material

Below is the link to the electronic supplementary material.

Supplementary Material 1 (DOCX 42.8 MB)

Supplementary Material 2 (DOCX 109 MB)

## Data Availability

The datasets generated and analyzed during the current study are available from the corresponding author upon reasonable request.

## Abbreviations

IS: Ischemic stroke
I/R: Ischemia-reperfusion
BBB: Blood-brain barrier
NMMHC IIA: Non-muscle myosin heavy chain IIA
*Myh9*^*ECKD*^: Endothelial-specific NMMHC IIA conditional knockdown
*Myh9*^*iECKI*^: NMMHC IIA-inducible endothelial conditional knock-in
TJs: Tight junction proteins
MCAO/R: Middle cerebral artery occlusion/reperfusion
OGD/R: Oxygen-glucose deprivation/re-oxygenation
rCCA: Right common carotid artery
rECA: Right external carotid artery
rICA: Right internal carotid artery
rMCA: Right middle cerebral artery
pBMECs: Primary brain microvascular endothelial cells
bEnd.3: Brain-derived endothelial cells
TTC: 2,3,5-Triphenyl tetrazolium chloride
EB: Evans blue
Co-IP: Co-immunoprecipitation
MMP: Matrix metalloproteinase
