# Supplementary material for: The role of ROCK1/MLC/NMMHC IIA-actin signaling in ischemic stroke-induced blood-brain barrier disruption: implications for therapeutic intervention
Source: Cell Mol Life Sci. 2025 Oct 30;82(1):373. doi: 10.1007/s00018-025-05808-4 (PMC12575896; doi:10.1007/s00018-025-05808-4)
Supplement: Supplementary file 1 — Supplementary Material 1 (DOCX 42.8 MB) [file 18_2025_5808_MOESM1_ESM.docx]

**Fig. 1a ROCK1 + β-actin**

**
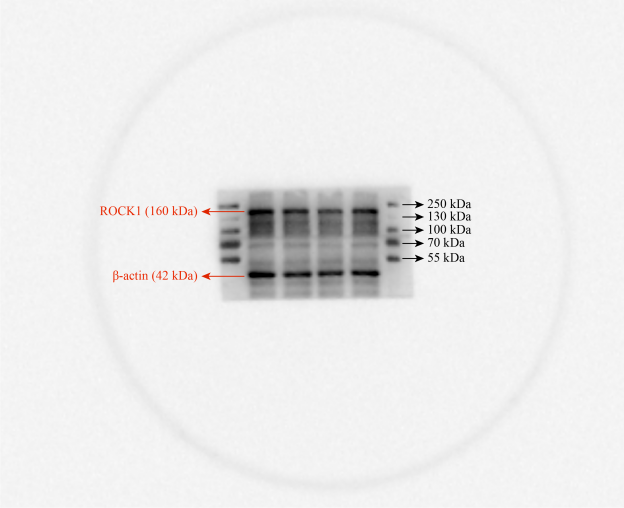

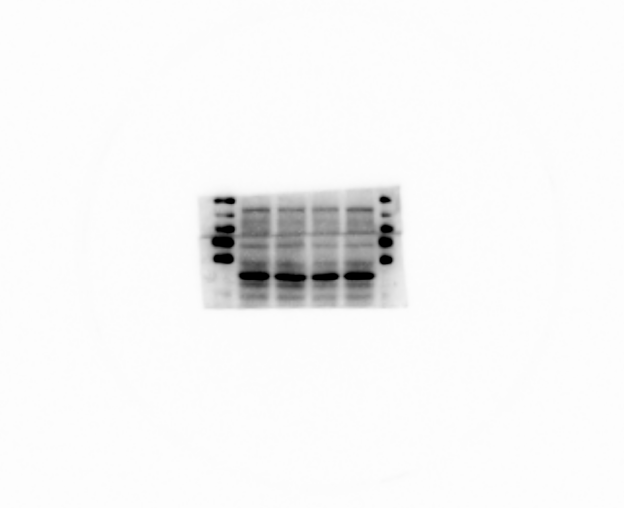
**

**
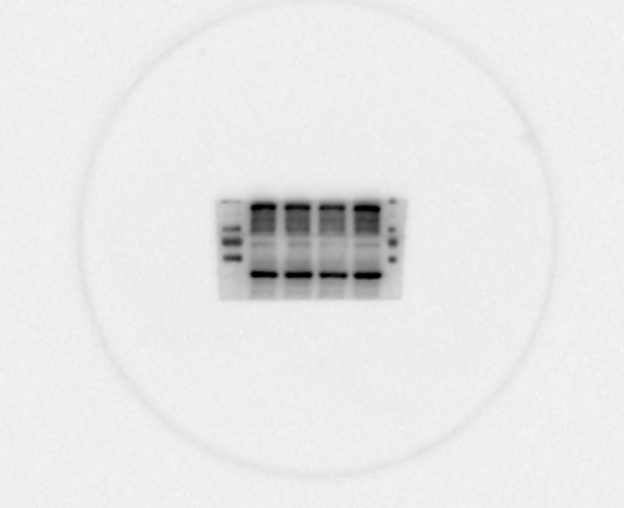

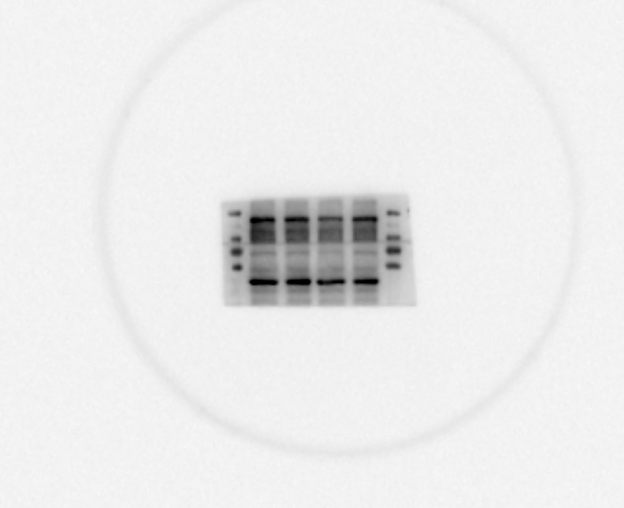
**

**
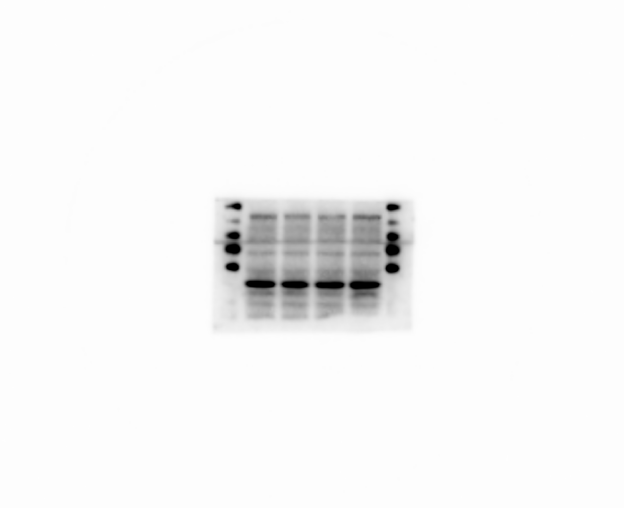
**

**Fig. 1b pMLC/MLC + β-actin**

1. **pMLC + β-actin**

**
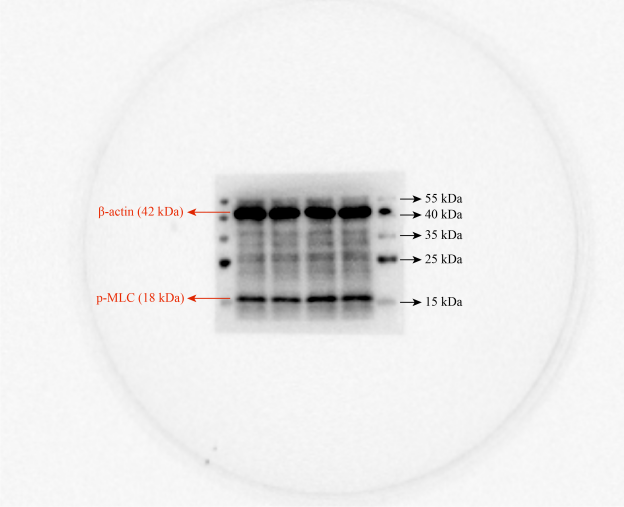

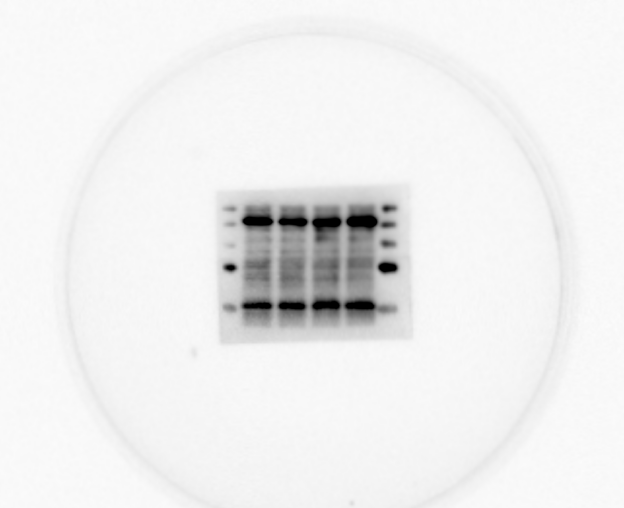
**

**
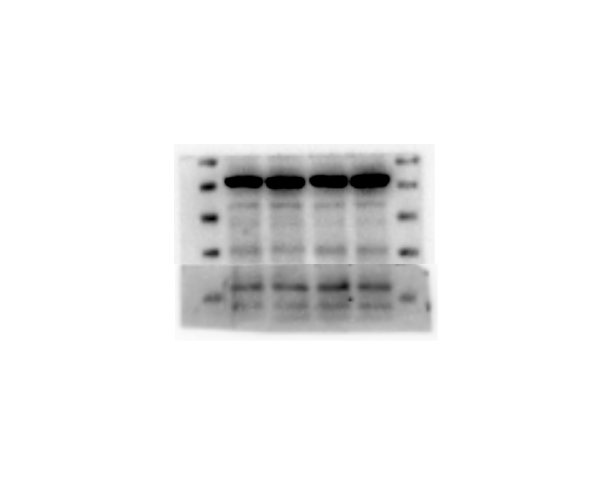

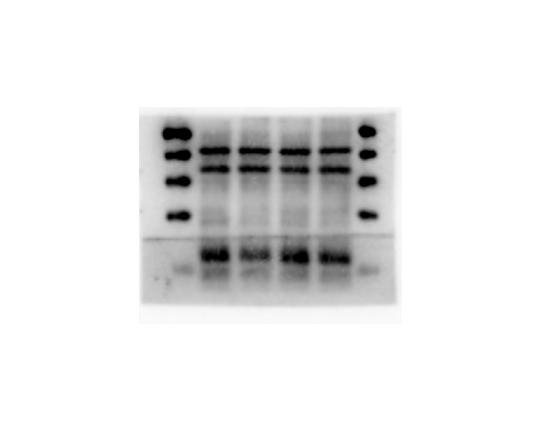
**

**
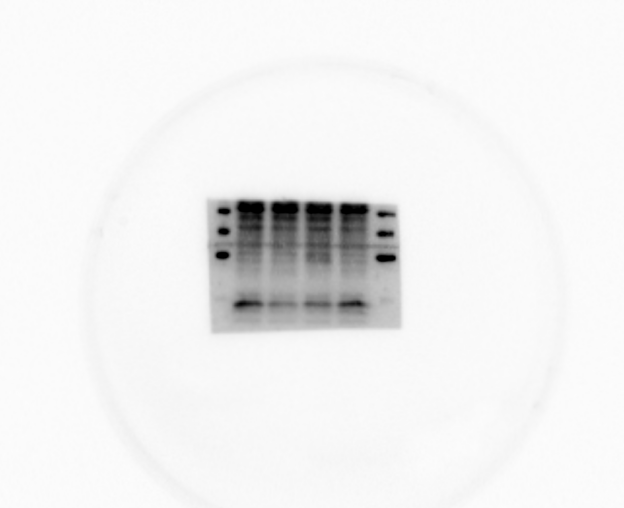
**

1. **MLC + β-actin**

**
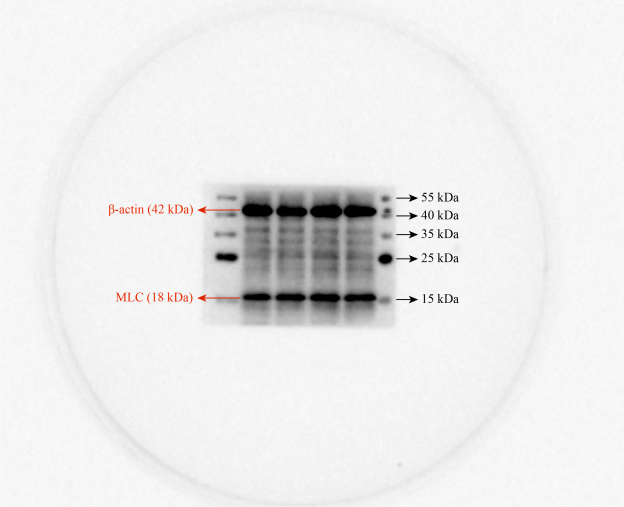

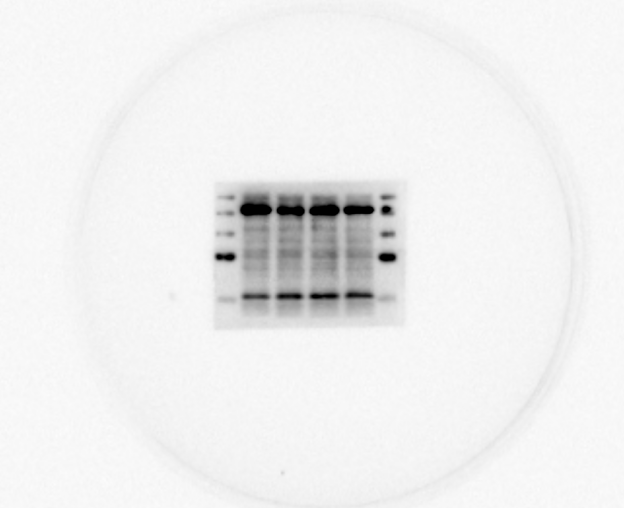
**

**
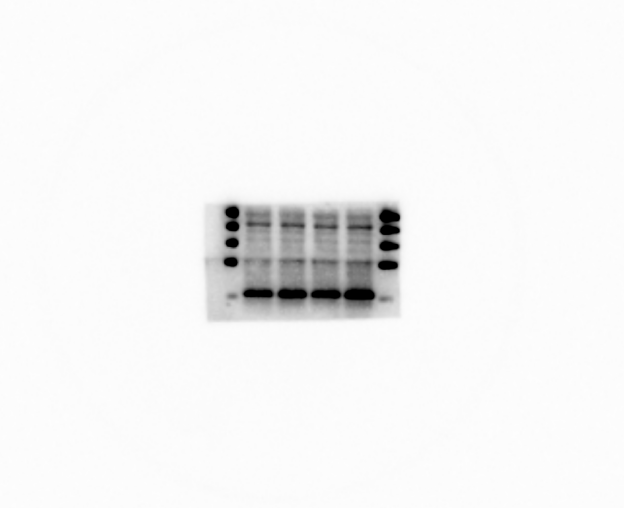

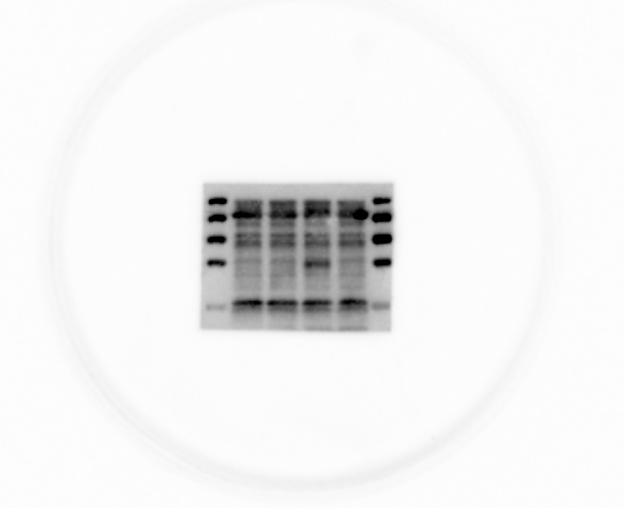
**

**
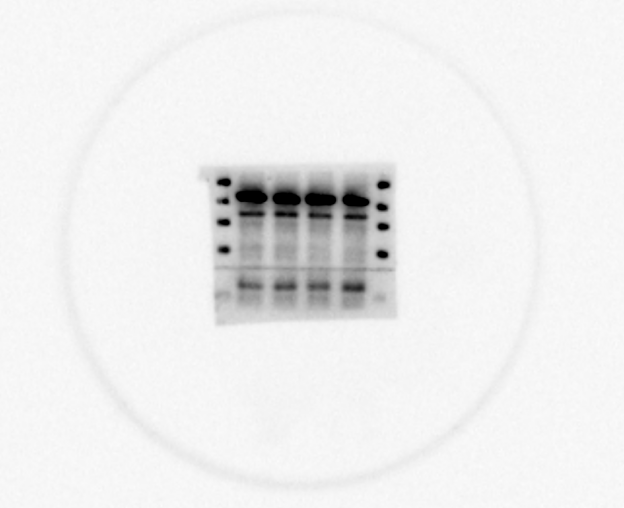
**

**Fig. 1c NMMHC IIA + β-actin**

**
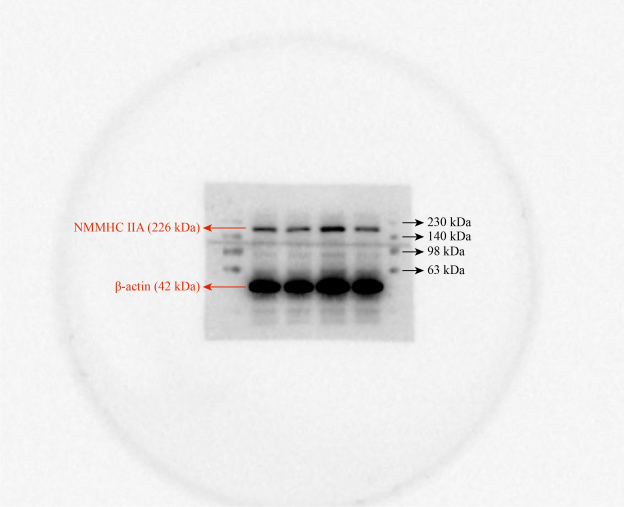

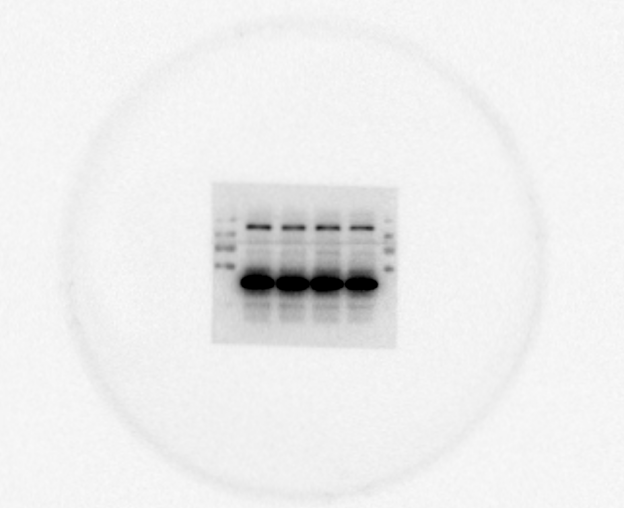
**

**
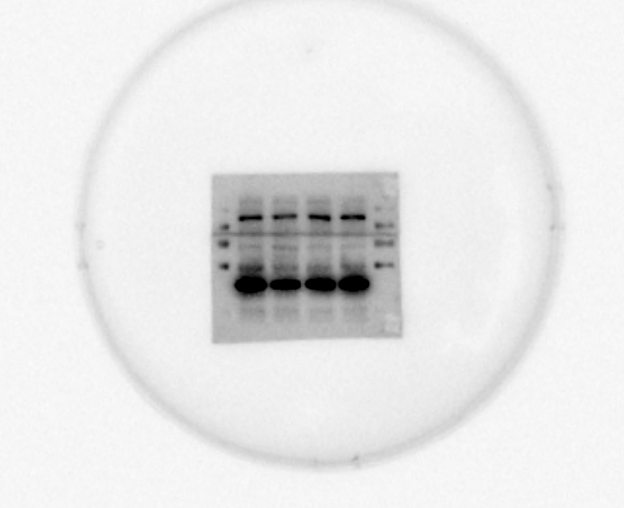

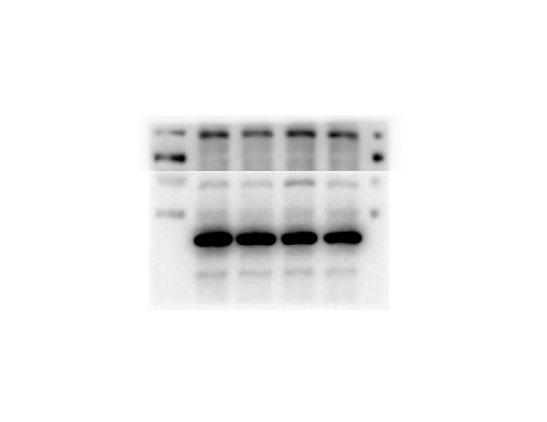

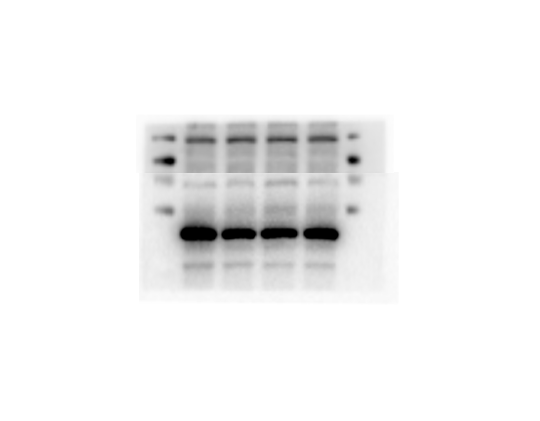
**

**Fig. 2a ROCK1 + β-actin**

**
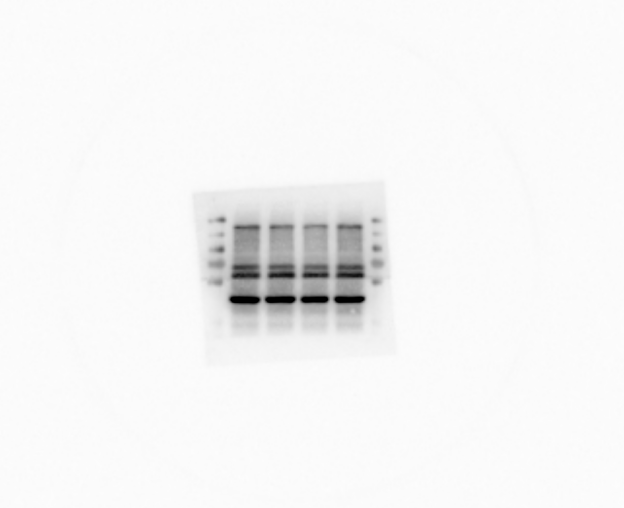

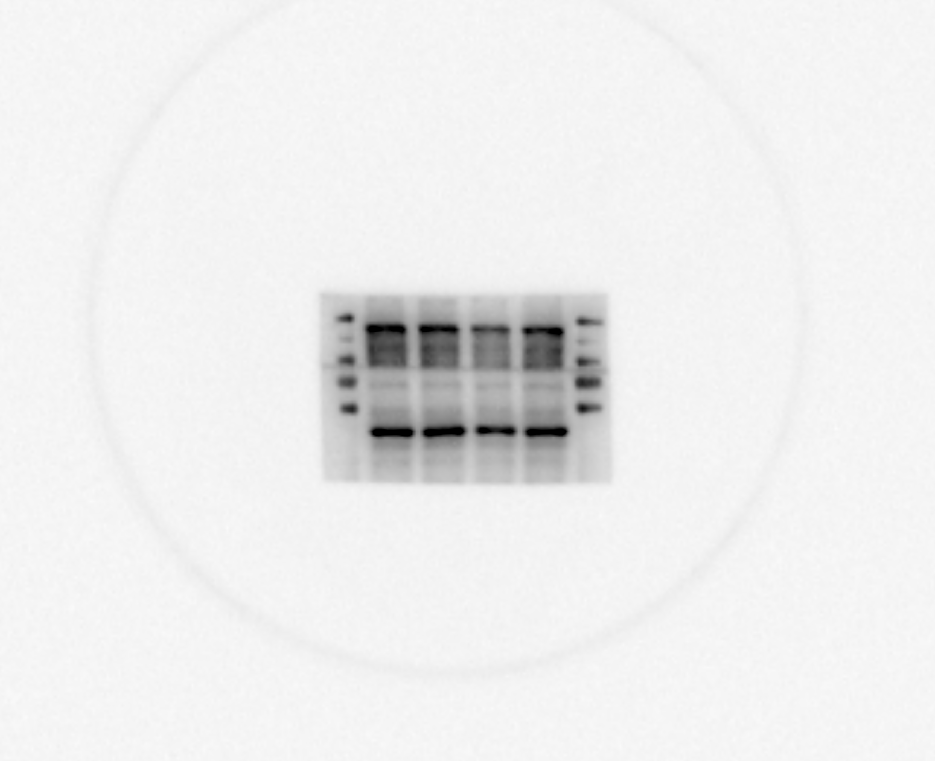

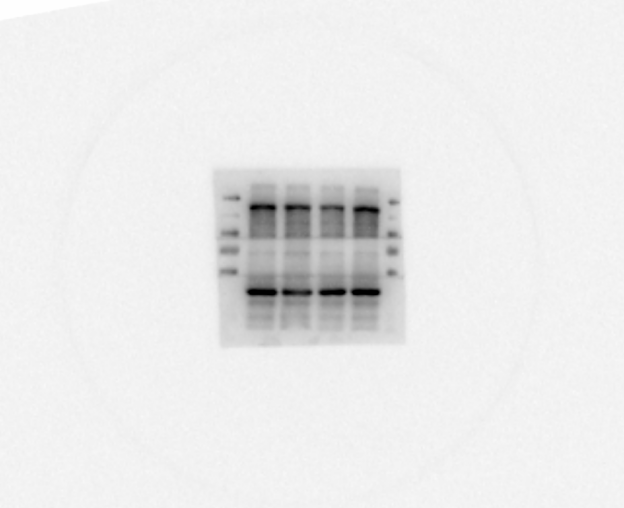

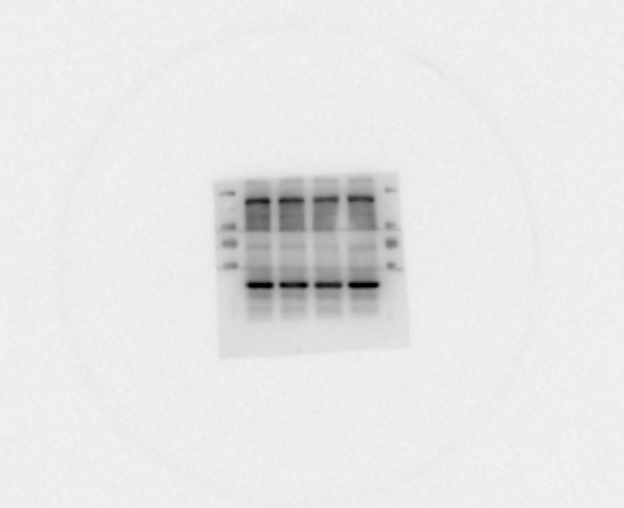

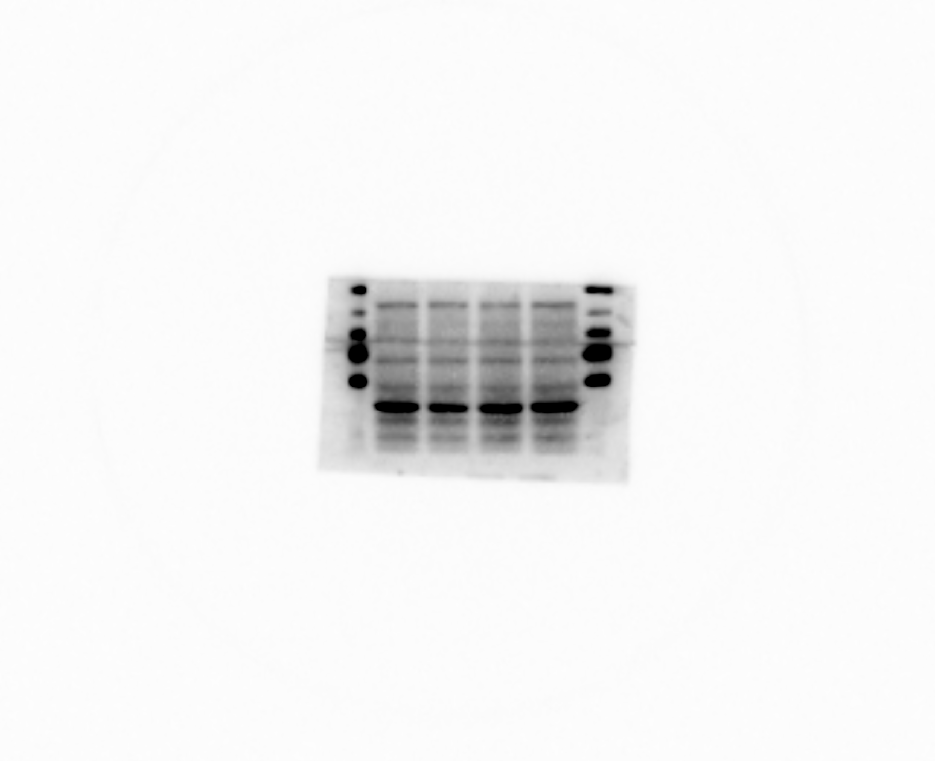
**

**Fig. 2b pMLC/MLC + β-actin**

1. **pMLC + β-actin**

**
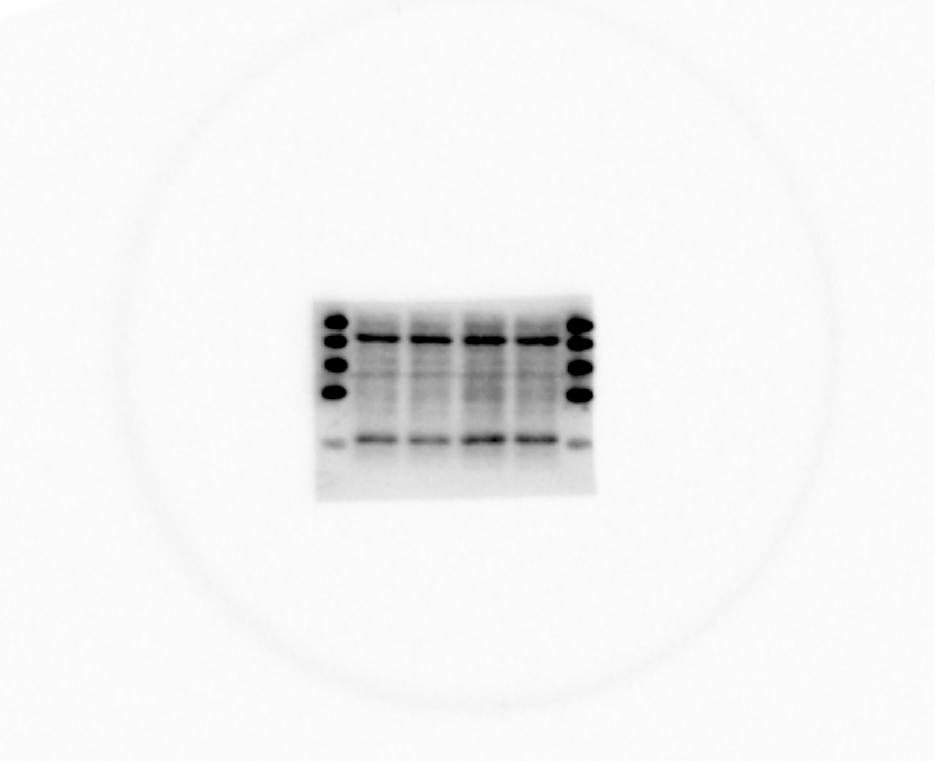

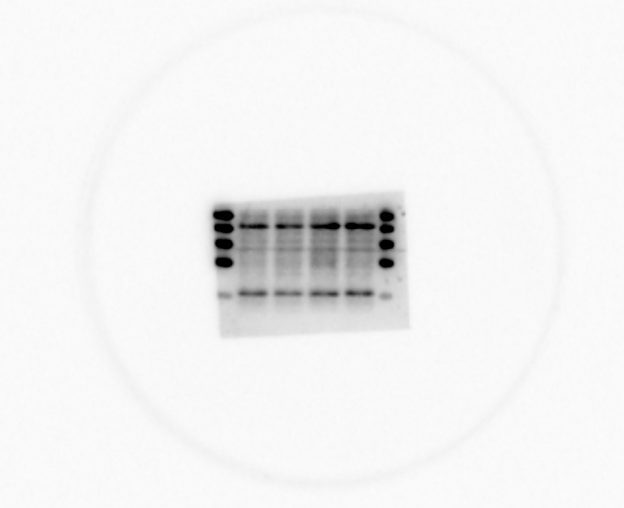
**

**
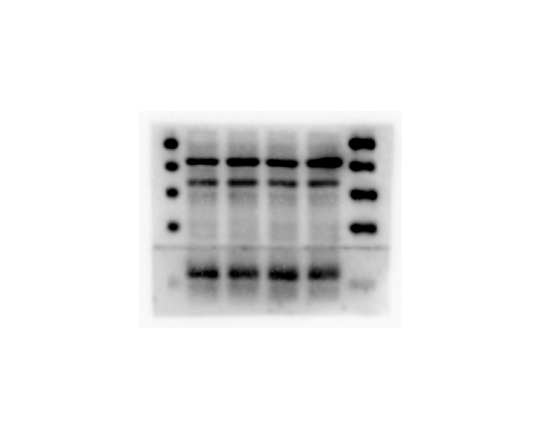

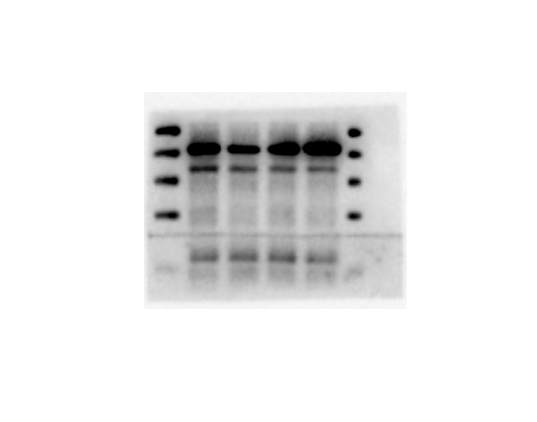

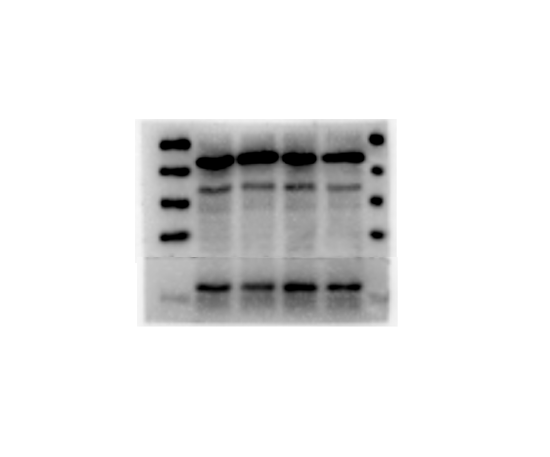
**

1. **MLC + β-actin**

**
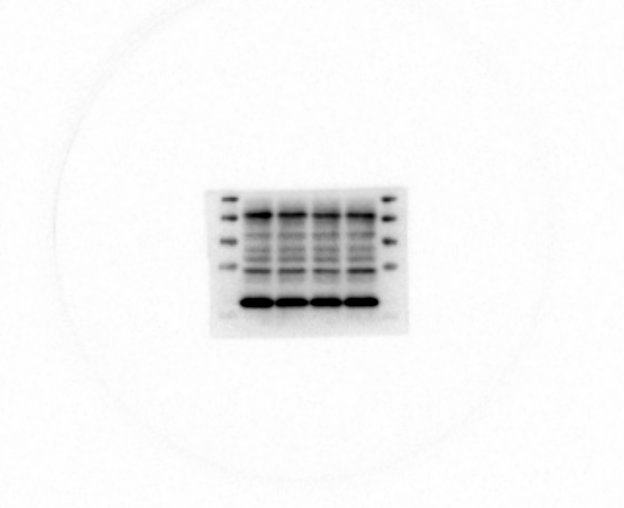

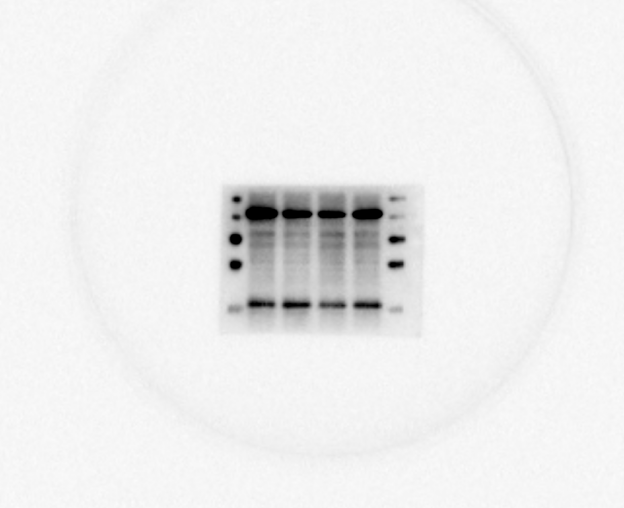
**

**
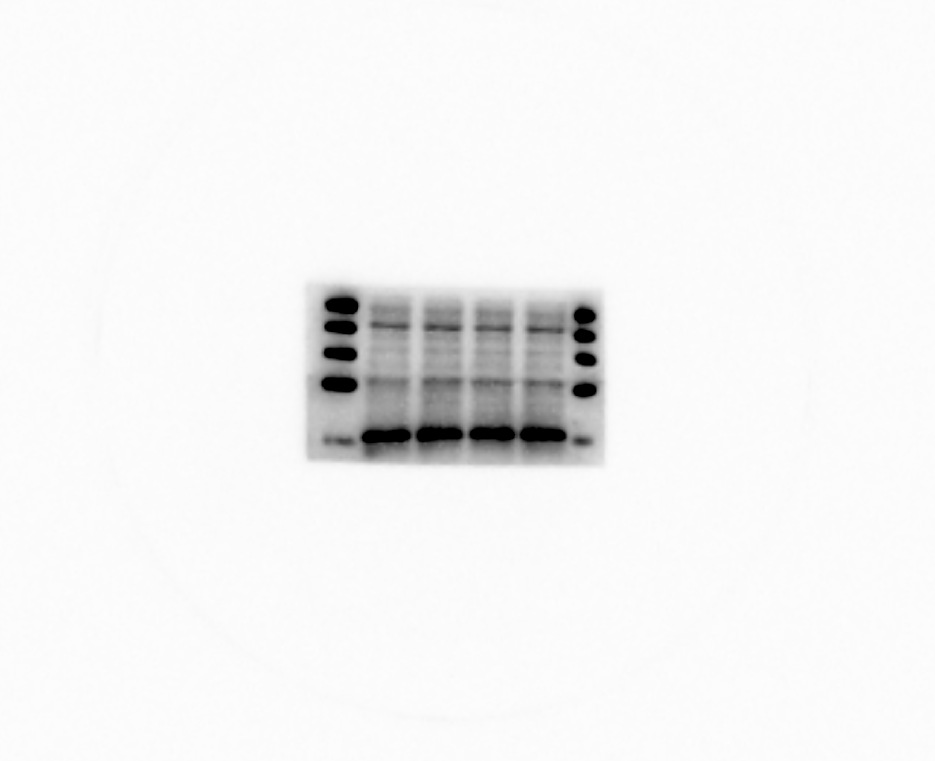

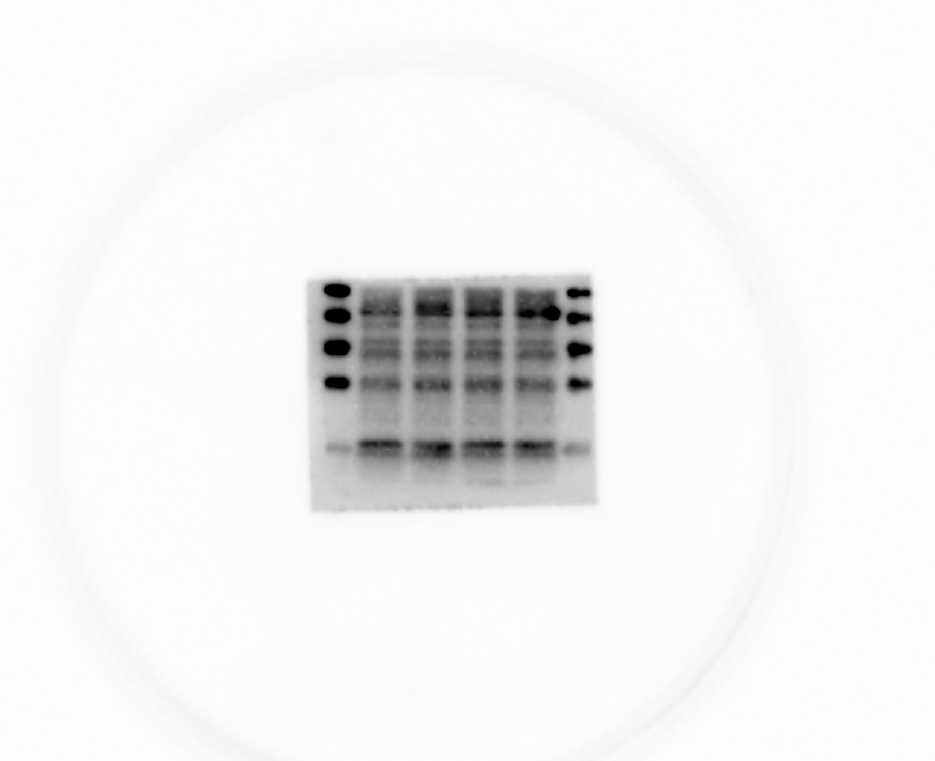

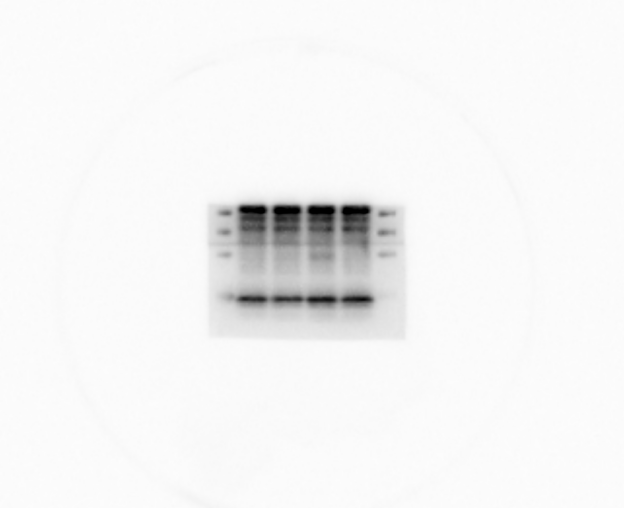
**

**Fig. 2c NMMHC IIA + β-actin**

**
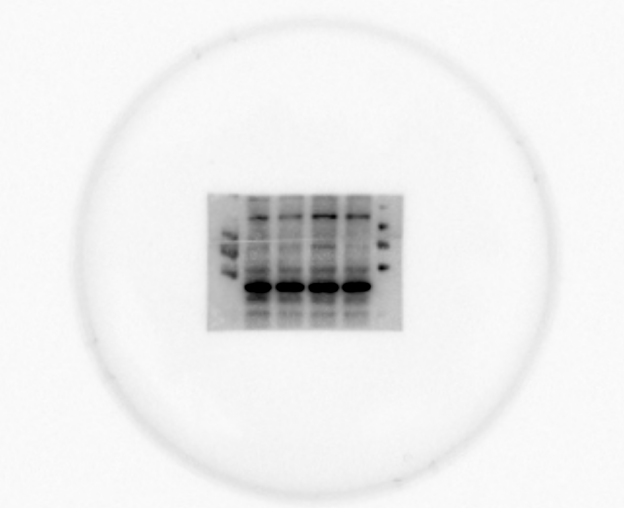

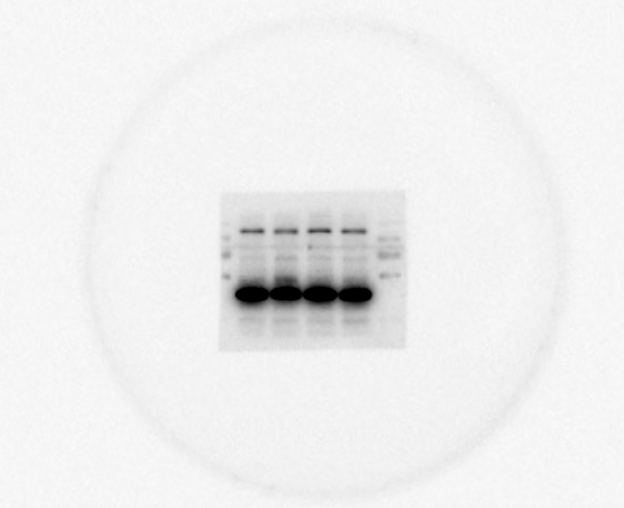
**

**
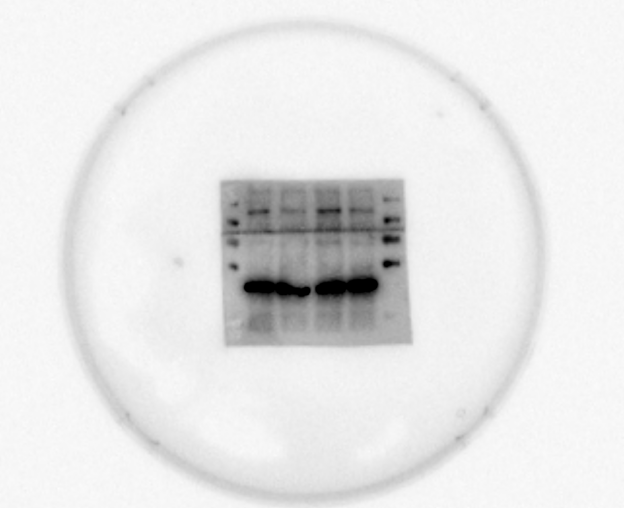

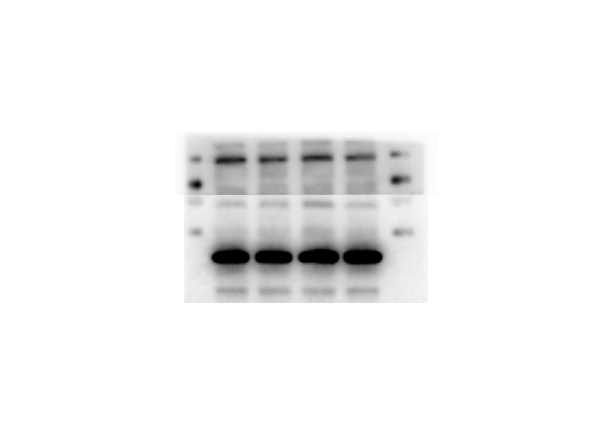
**

**
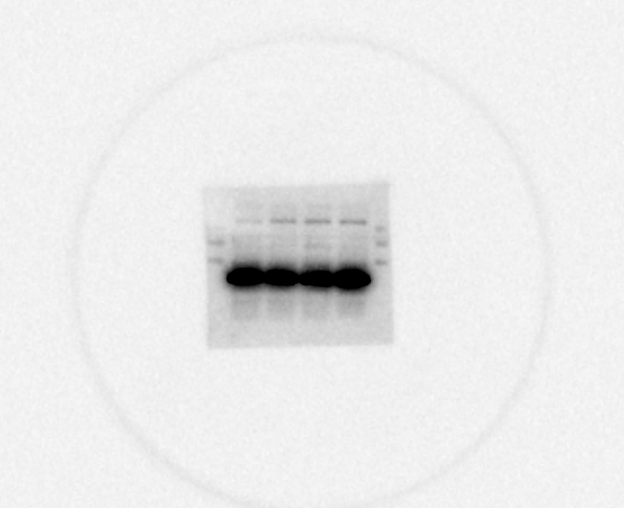
**

**Fig. 2e NMMHC IIA + F-actin (Co-IP)**

**
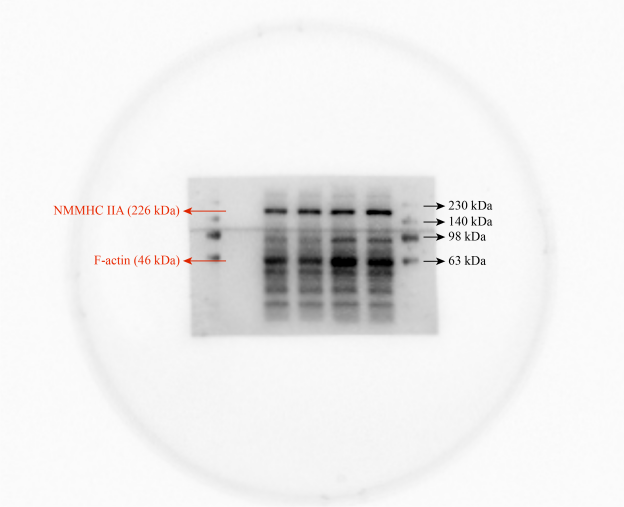

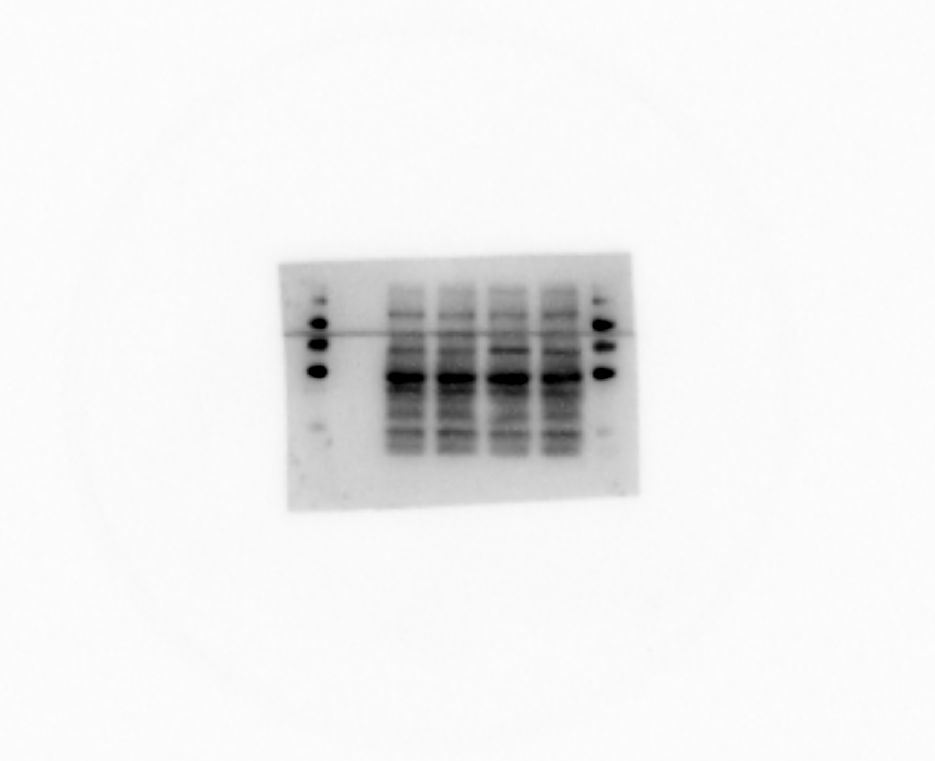
**

**
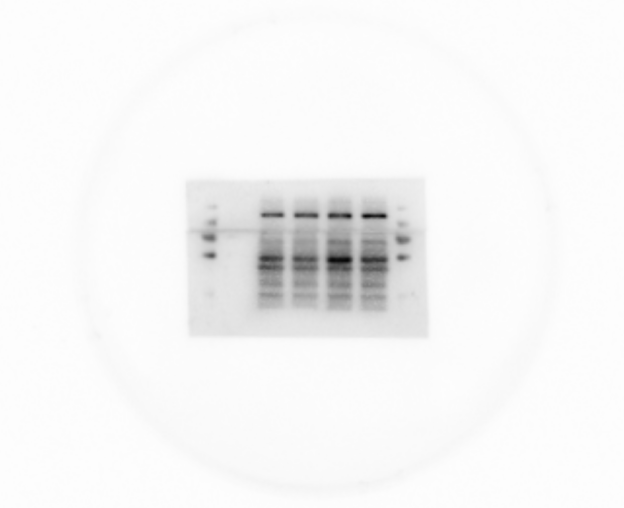

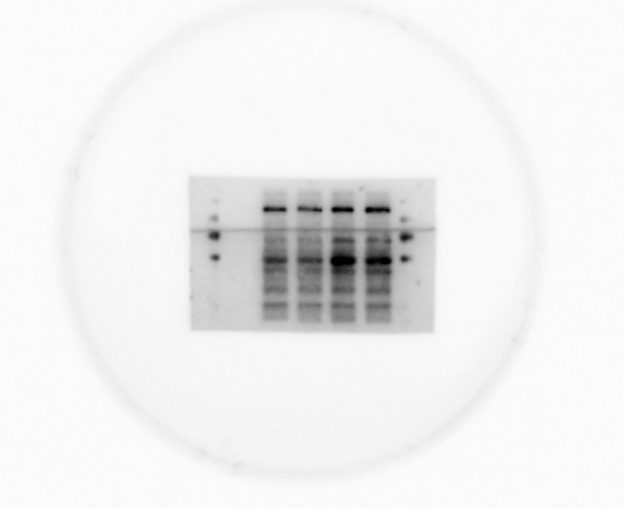
**

**
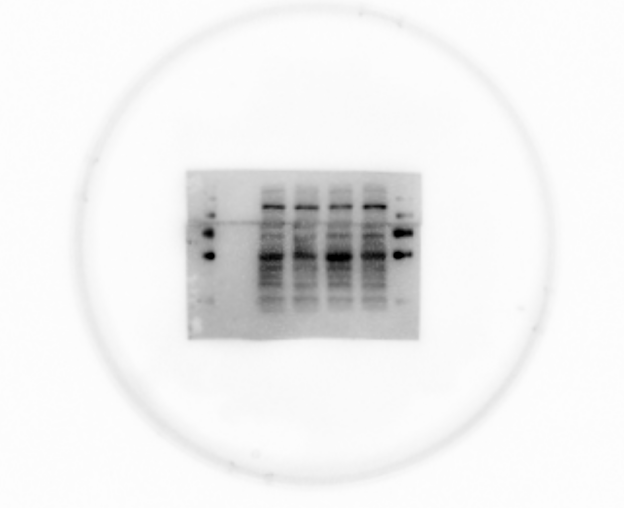
**

**Fig. 2f ZO-1 + α-Tubulin**

**
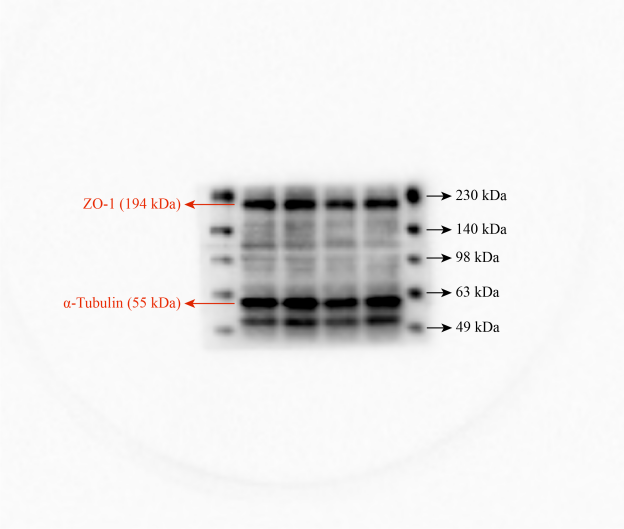

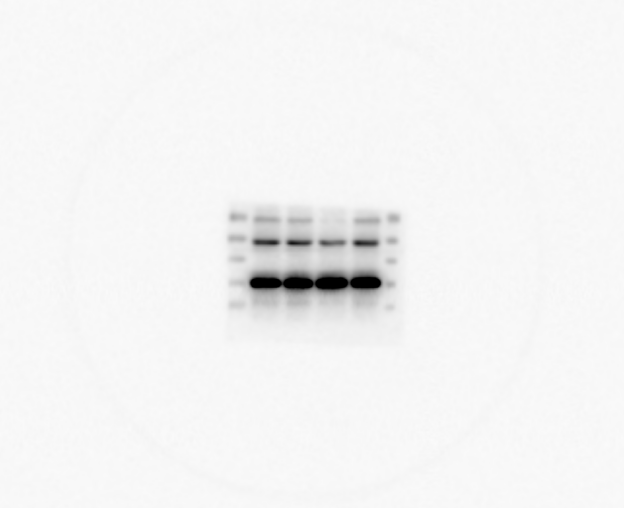
**

**
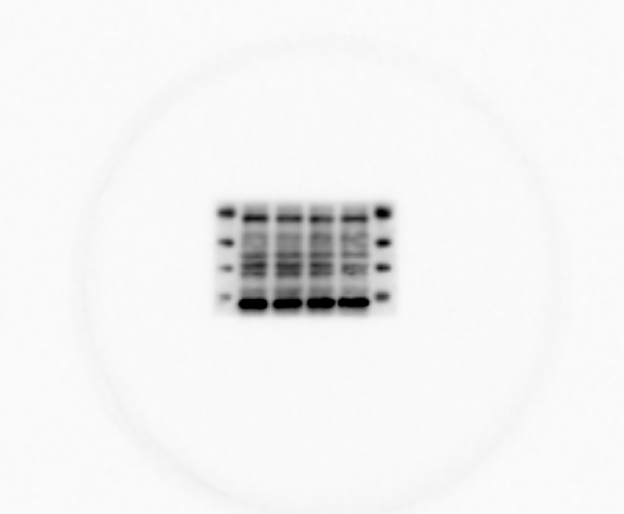

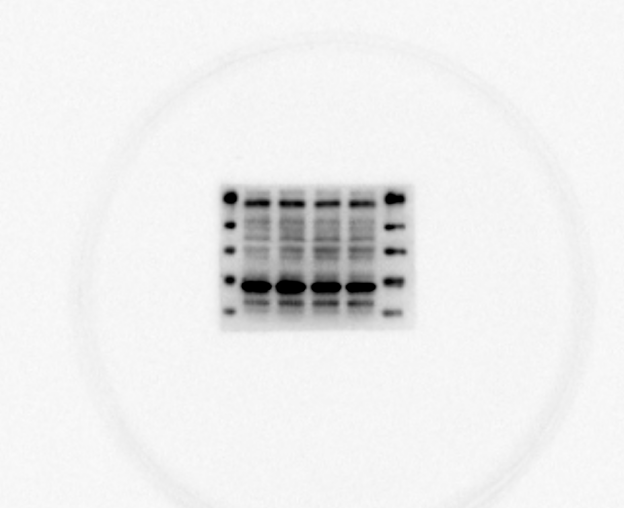
**

**
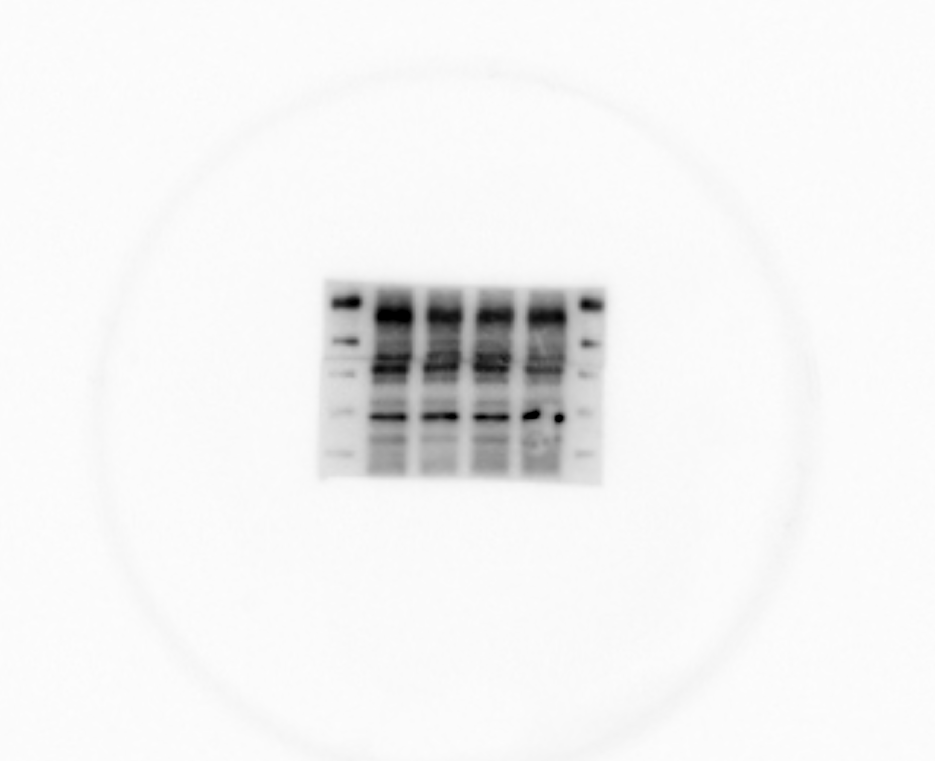
**

**Fig. 2g Occludin + β-actin**

**
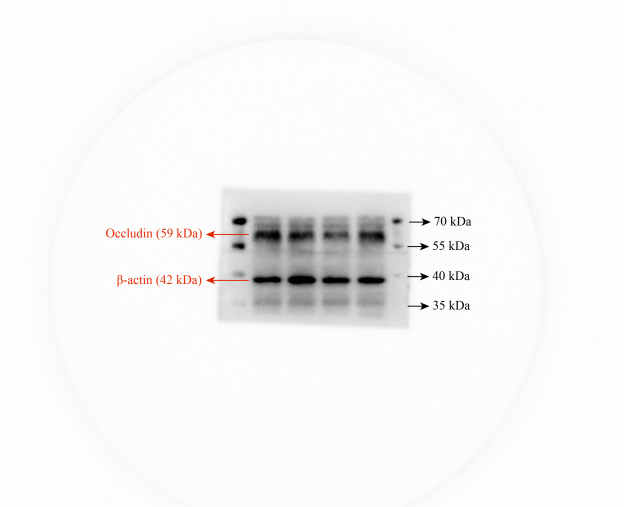

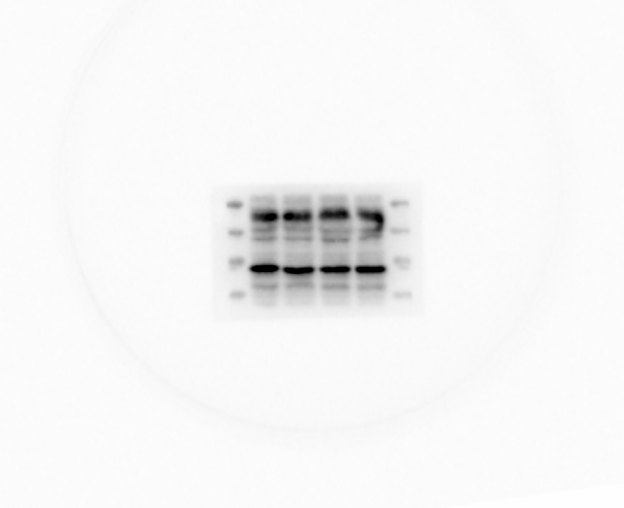
**

**
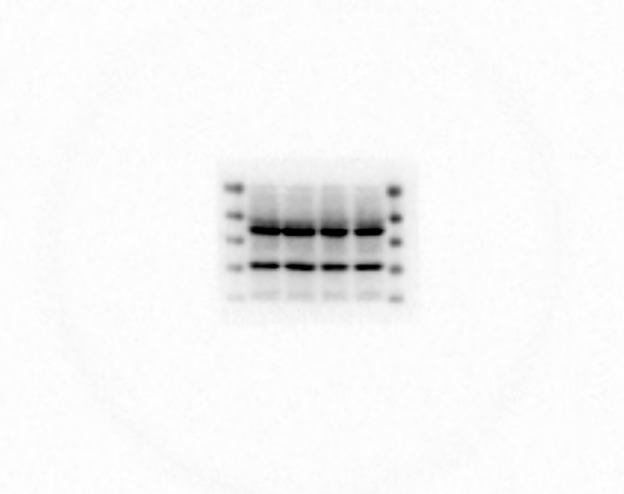

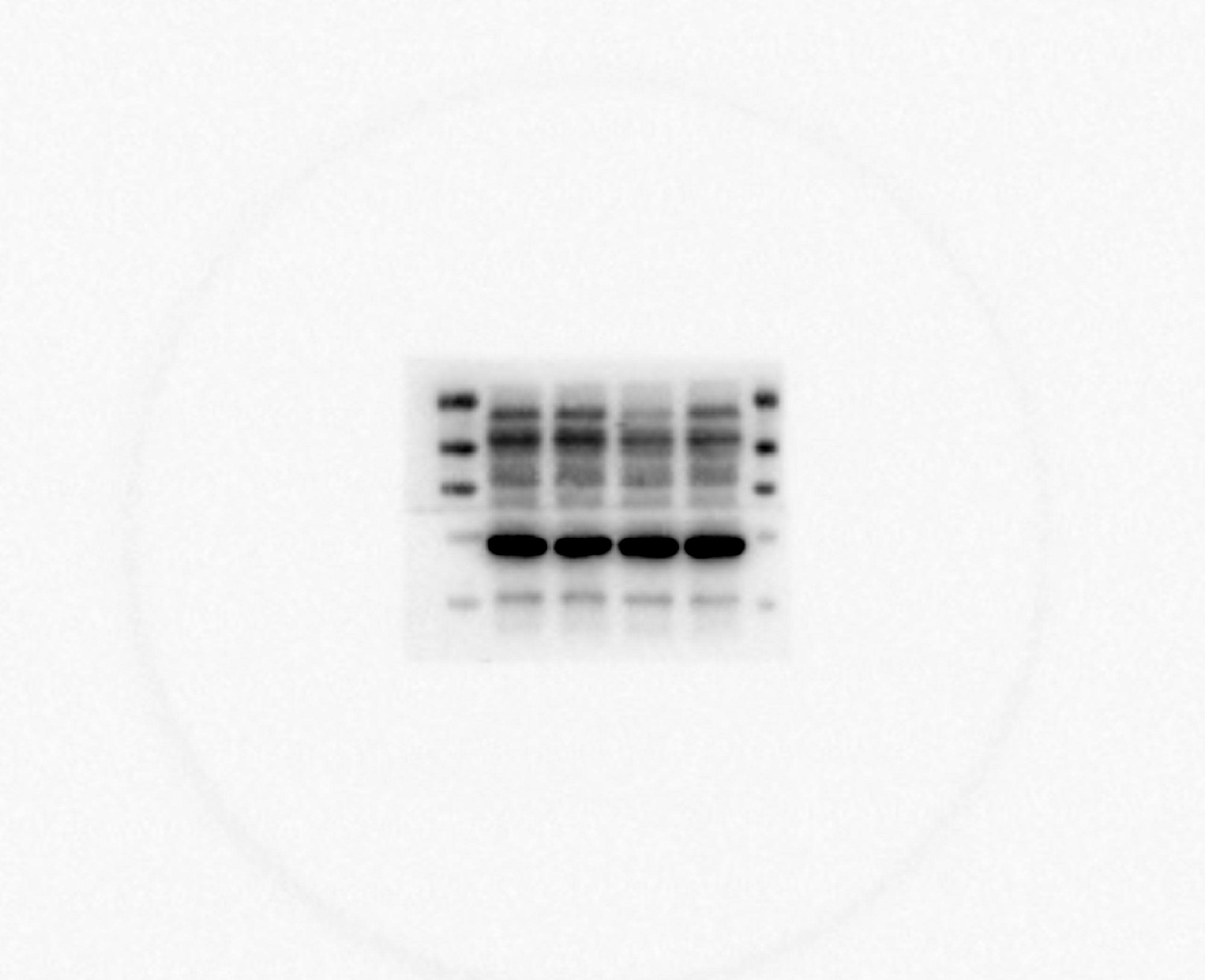
**

**
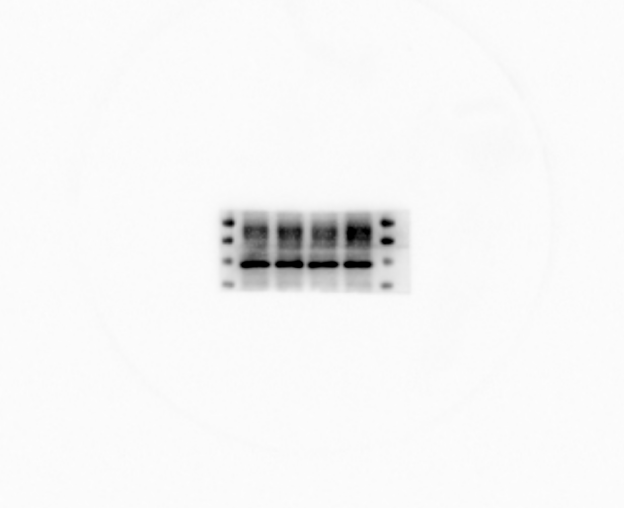
**

**Fig. 3f ZO-1 + α-Tubulin**

**
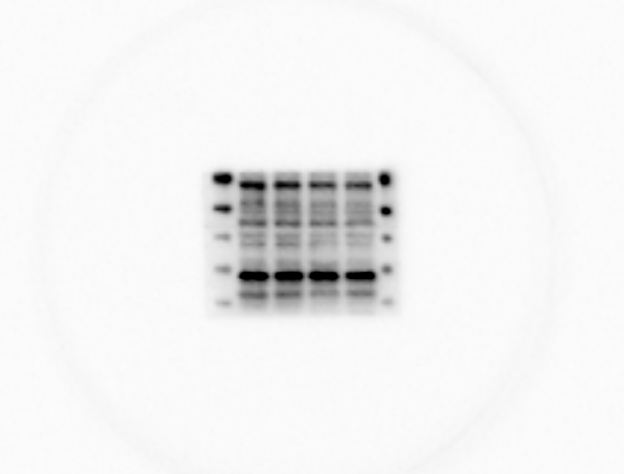

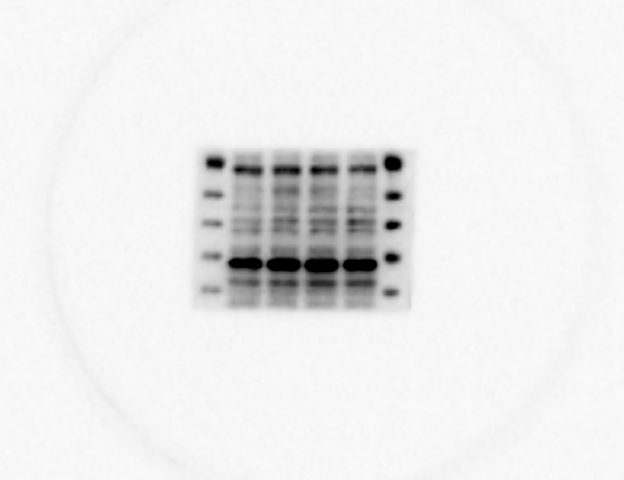
**

**
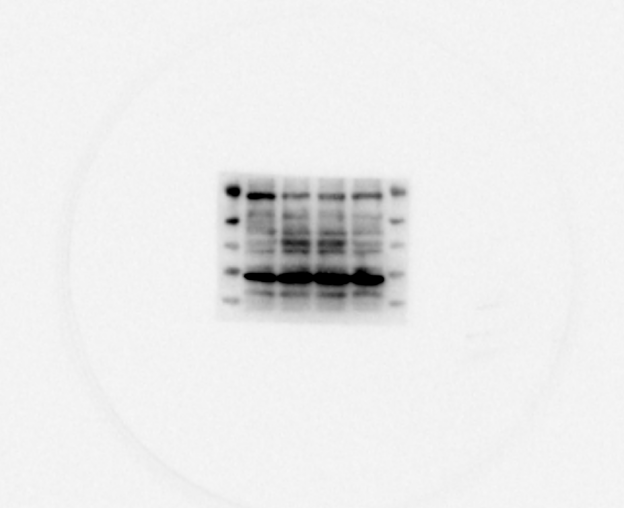

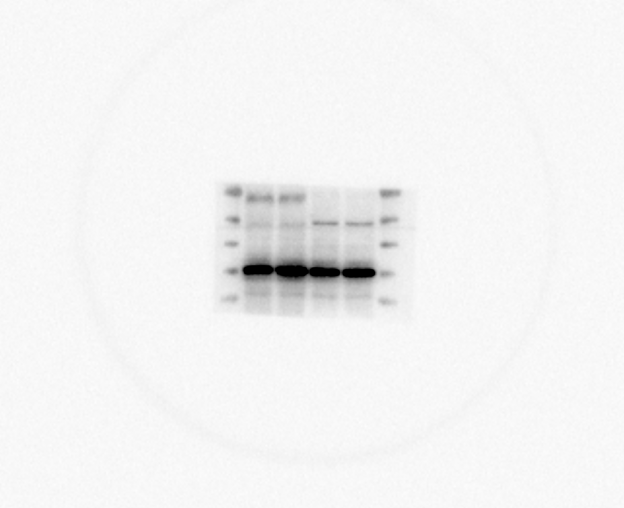
**

**
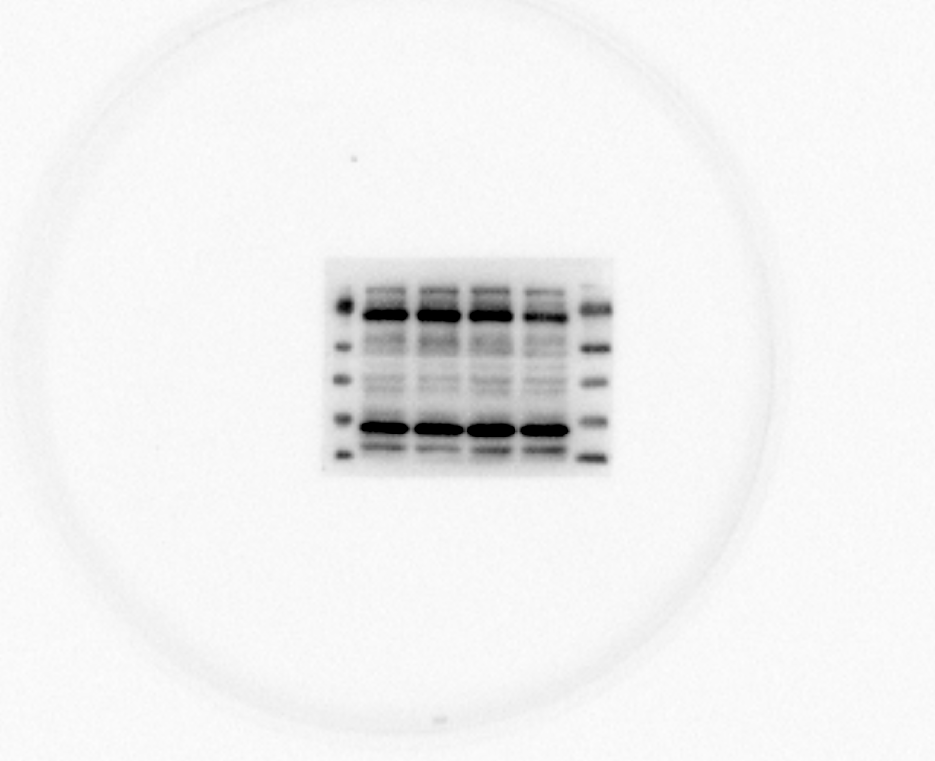
**

**Fig. 3g Occludin + β-actin**

**
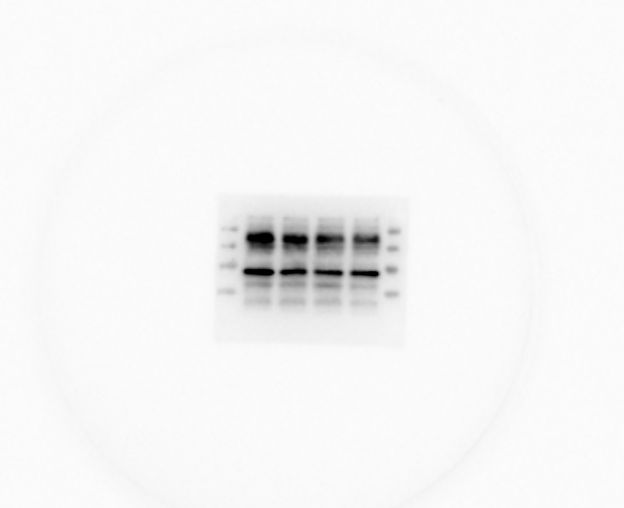

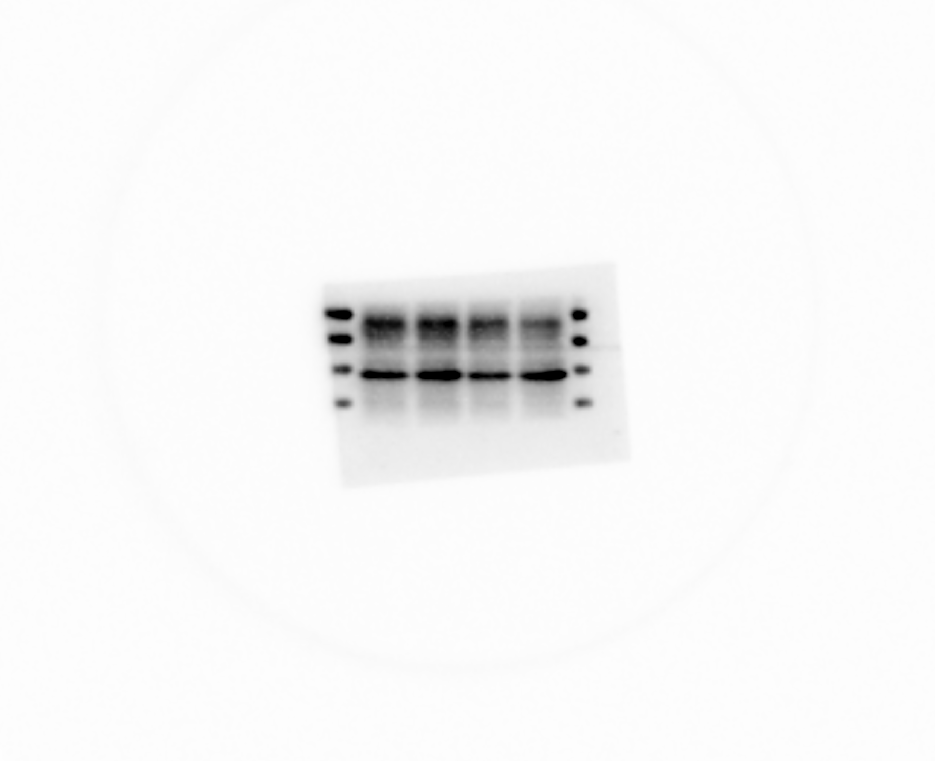
**

**
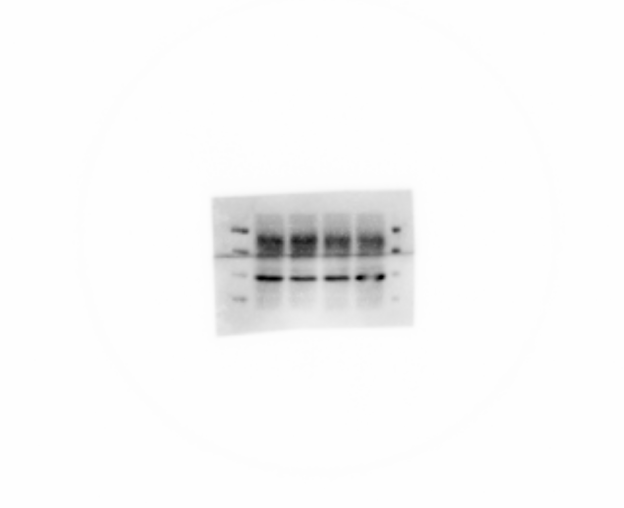

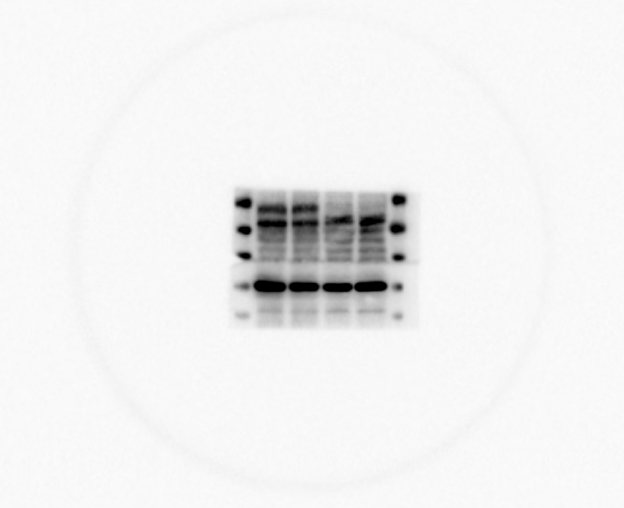
**

**
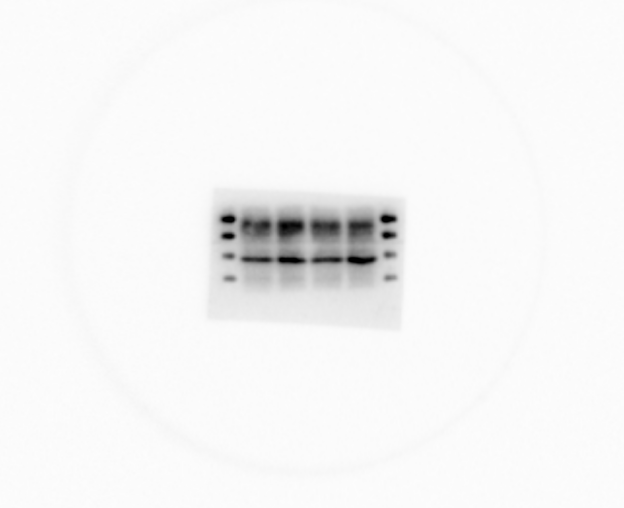
**

**Fig. 4a ROCK1 + β-actin**

**
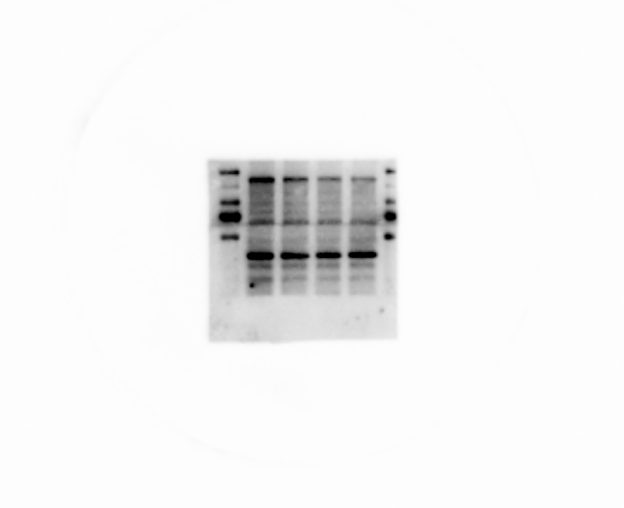

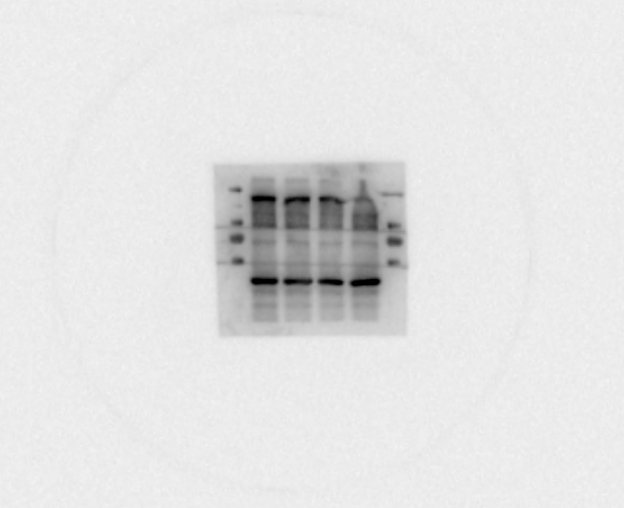
**

**
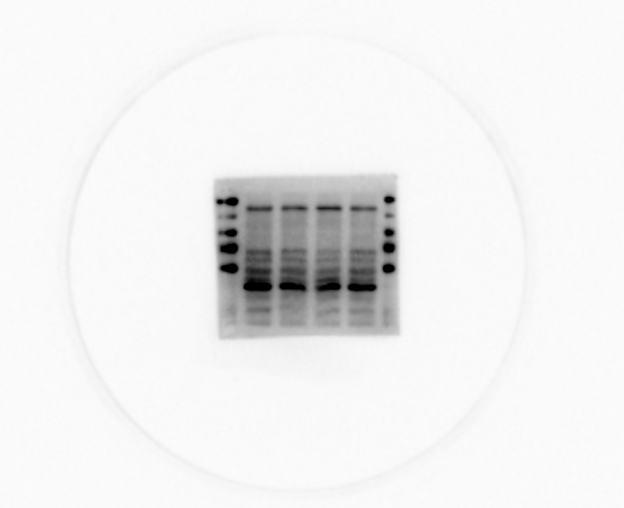

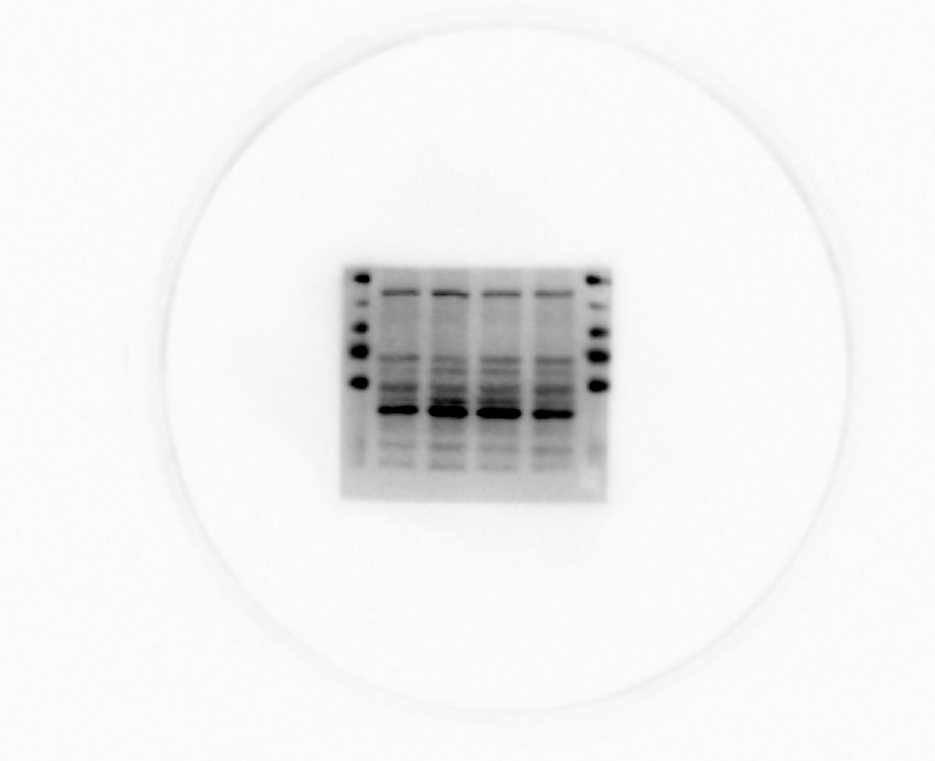
**

**
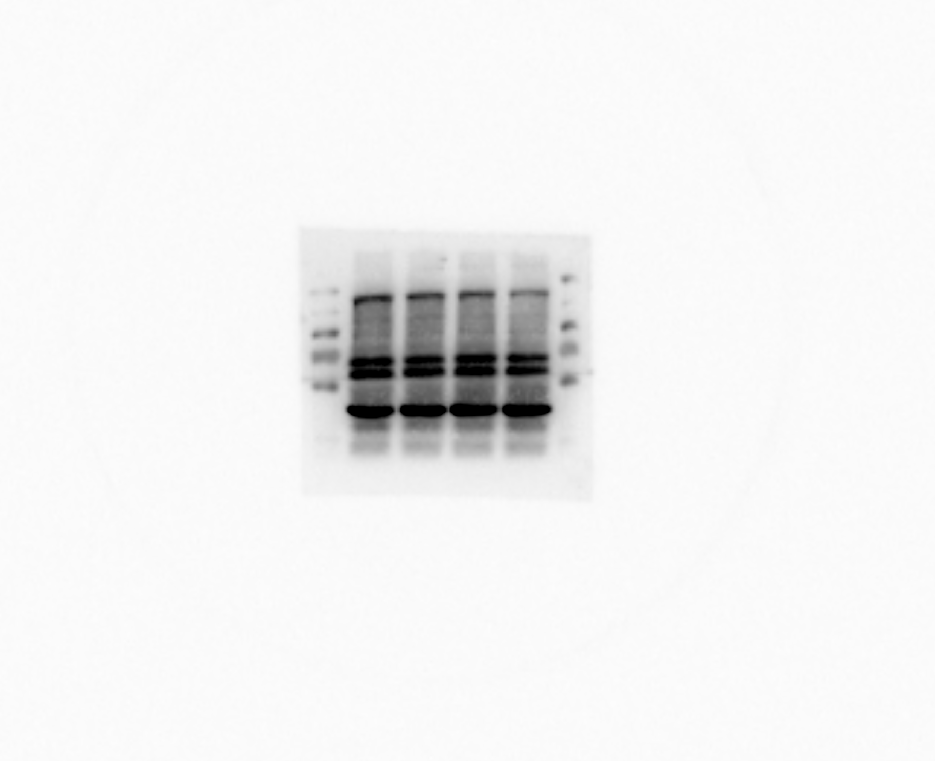
**

**Fig. 4b pMLC/MLC + β-actin**

1. **pMLC + β-actin**

**
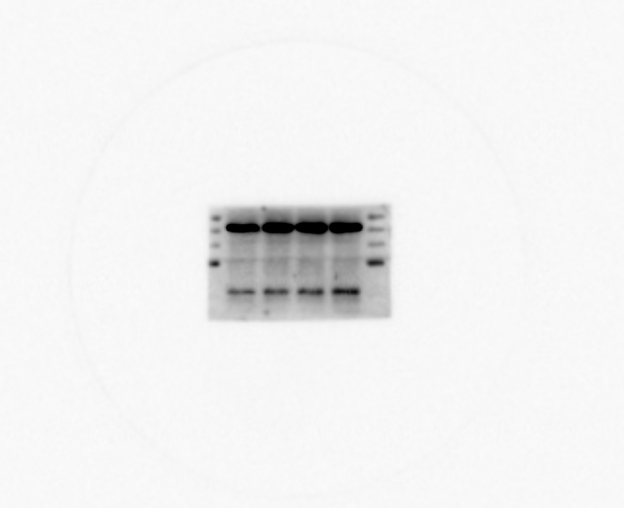

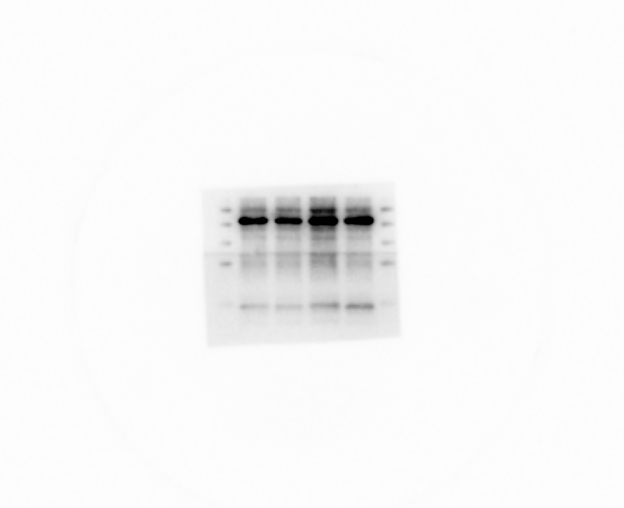
**

**
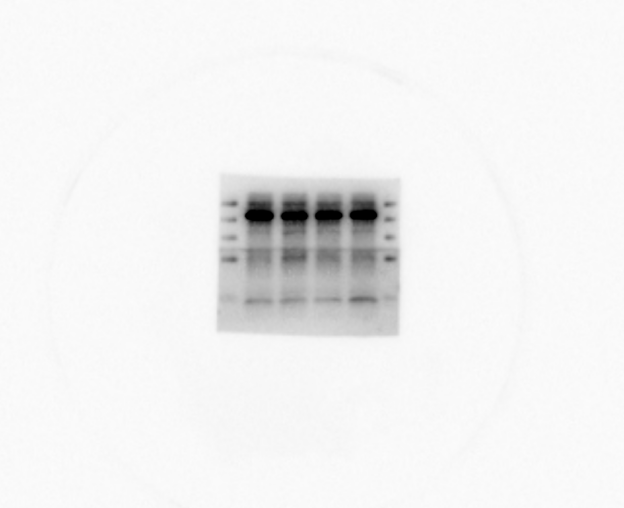

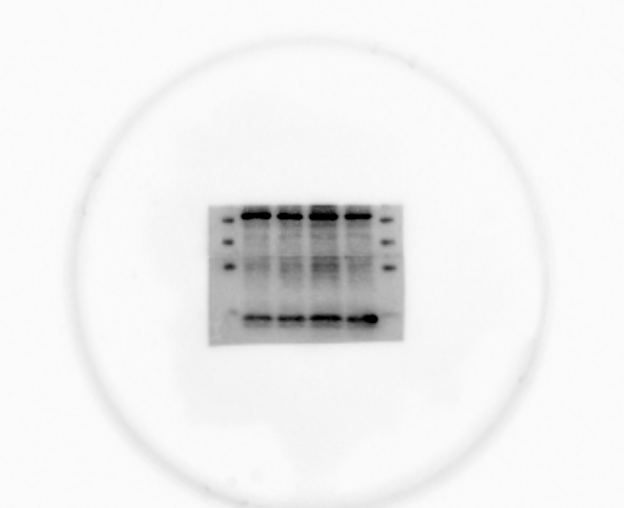
**

**
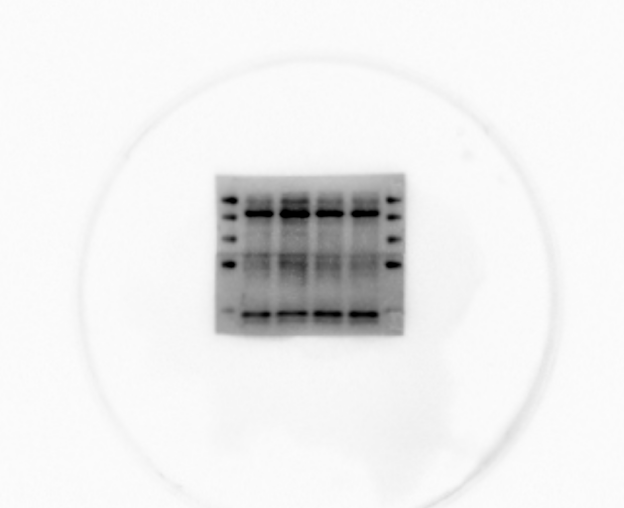
**

1. **MLC + β-actin**

**
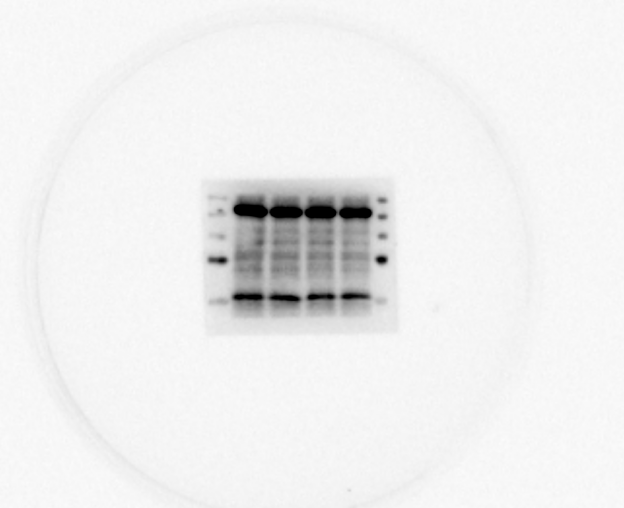

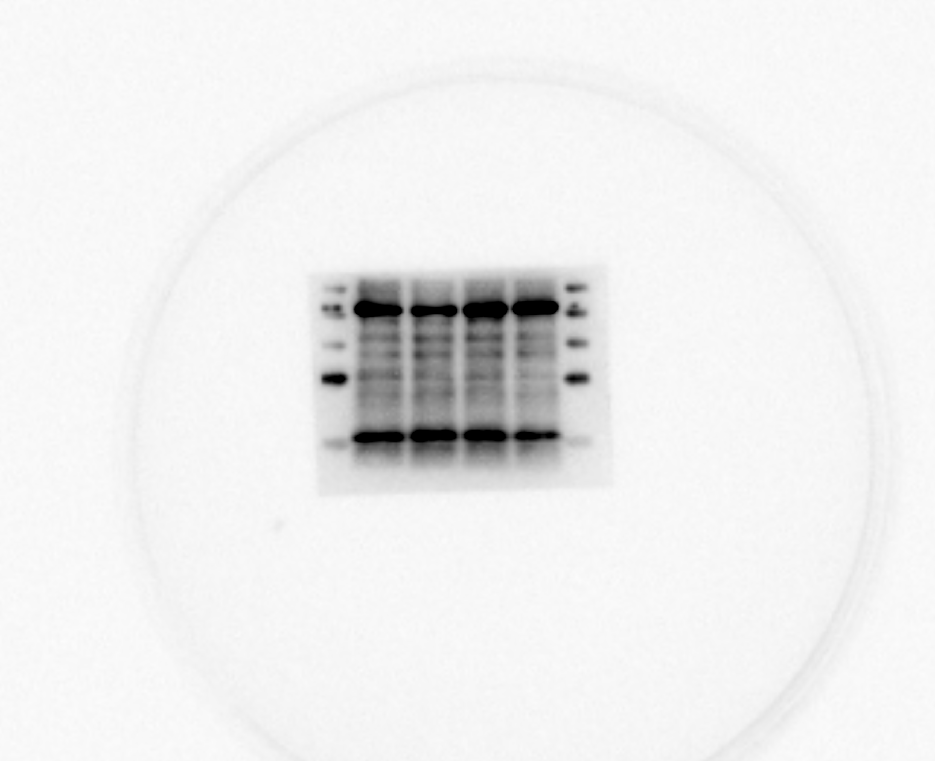
**

**
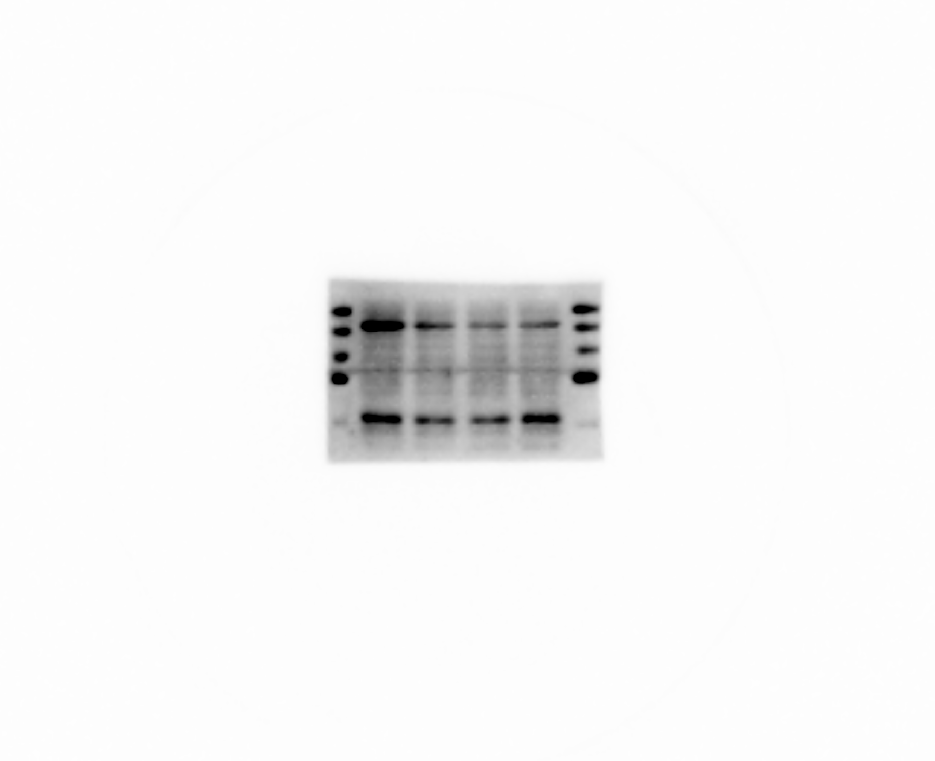

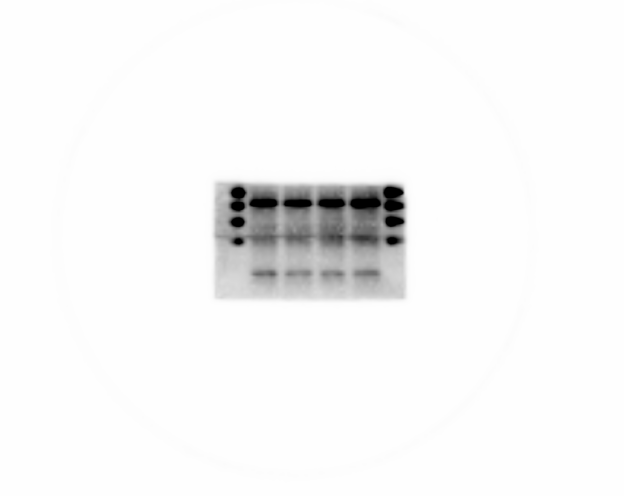
**

**
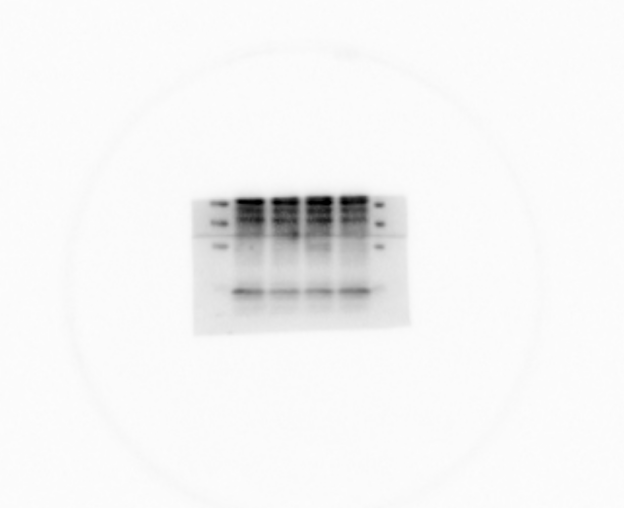
**

**Fig. 4c NMMHC IIA + β-actin**

**
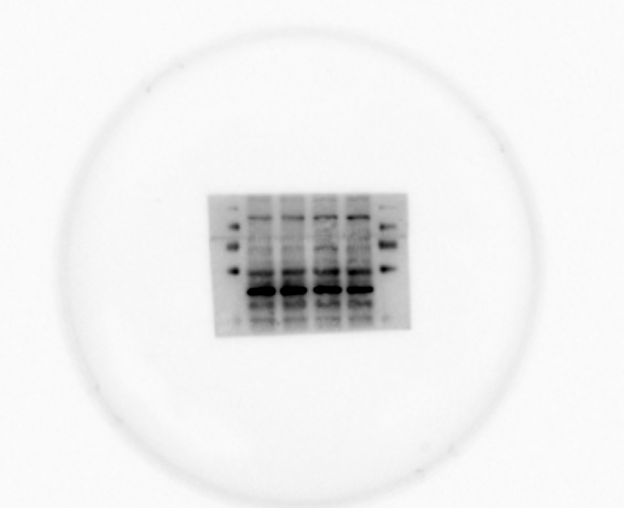

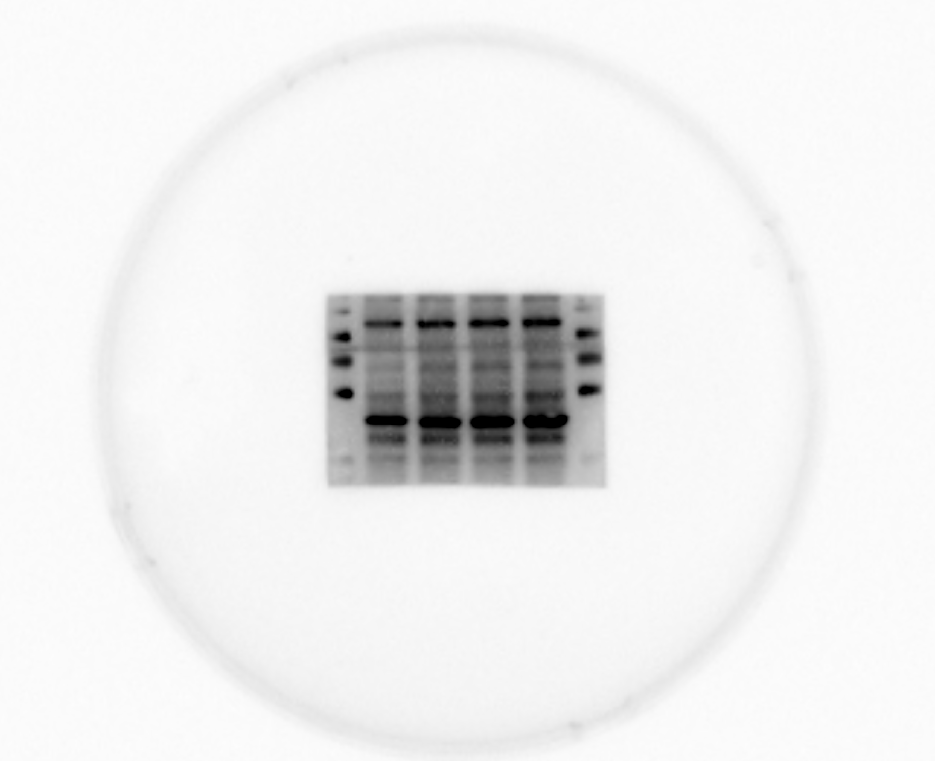
**

**
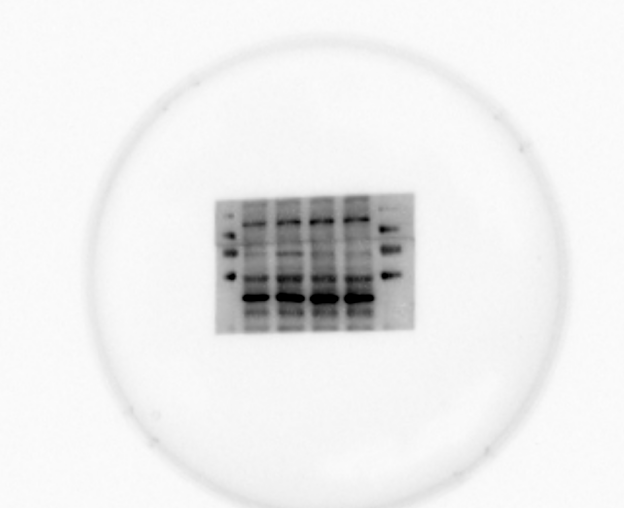

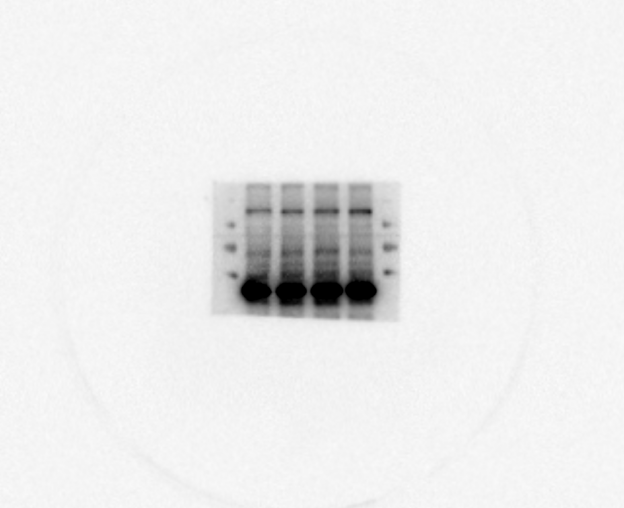
**

**
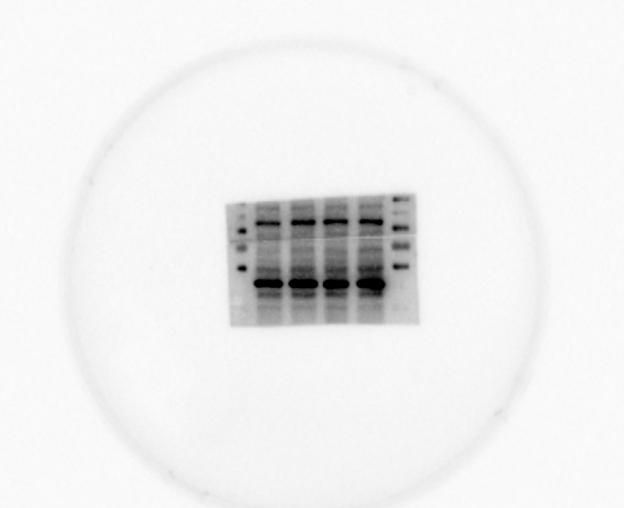
**

**Fig. 5f ZO-1 + α-Tubulin**

**
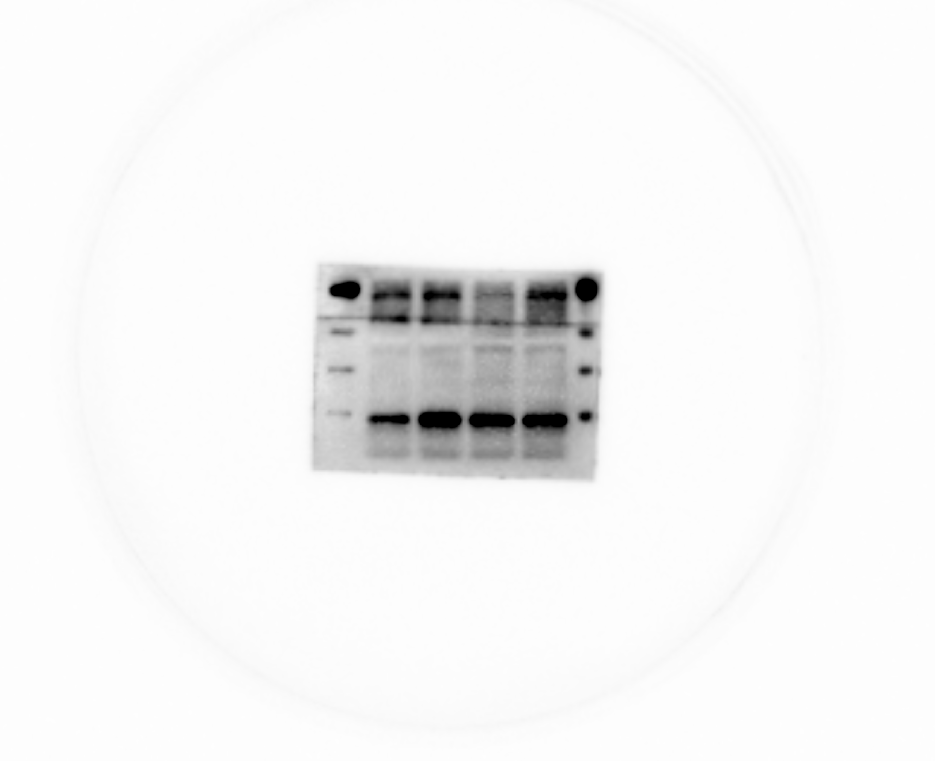

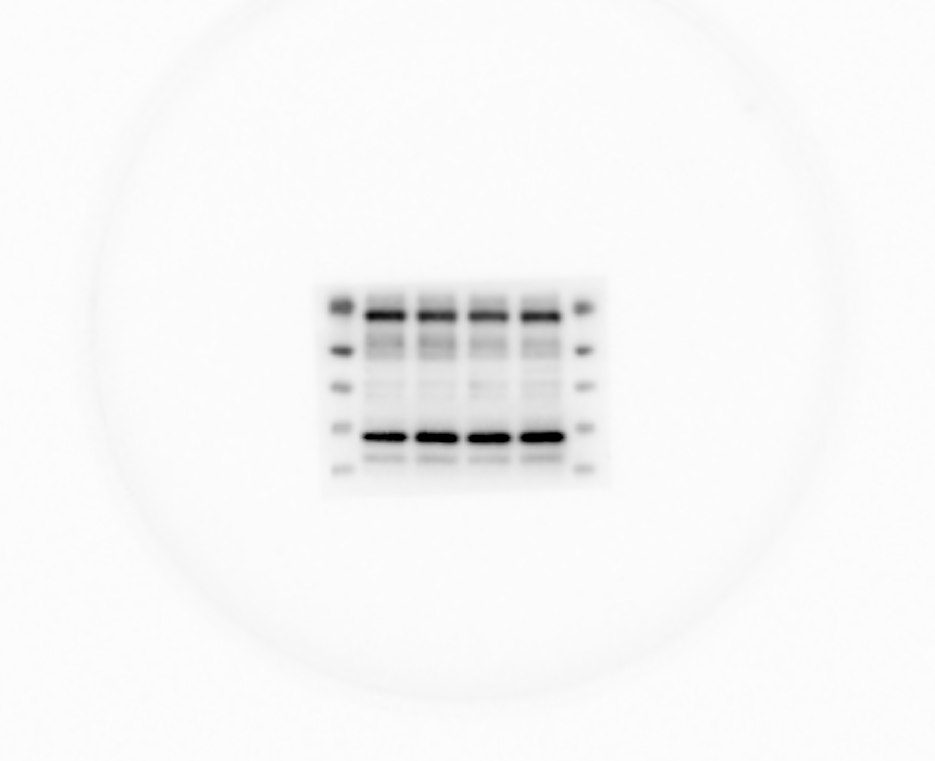
**

**
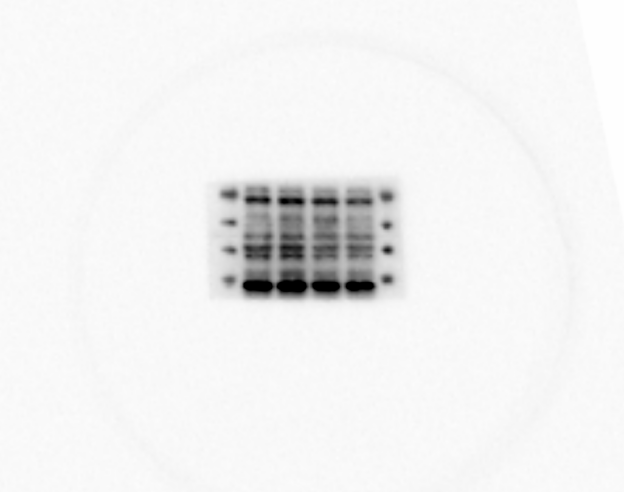

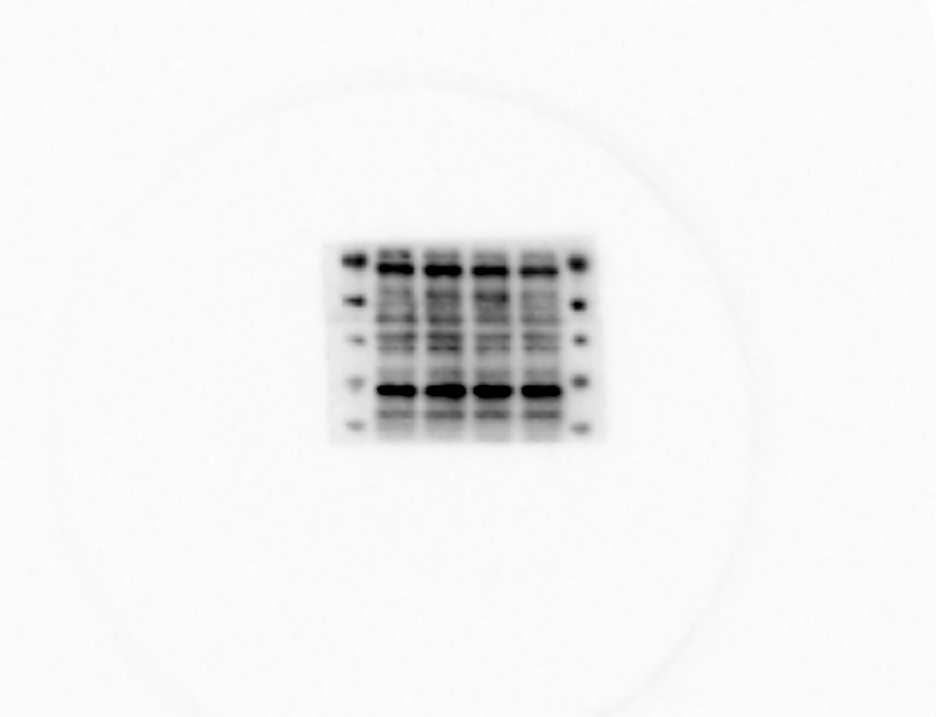
**

**
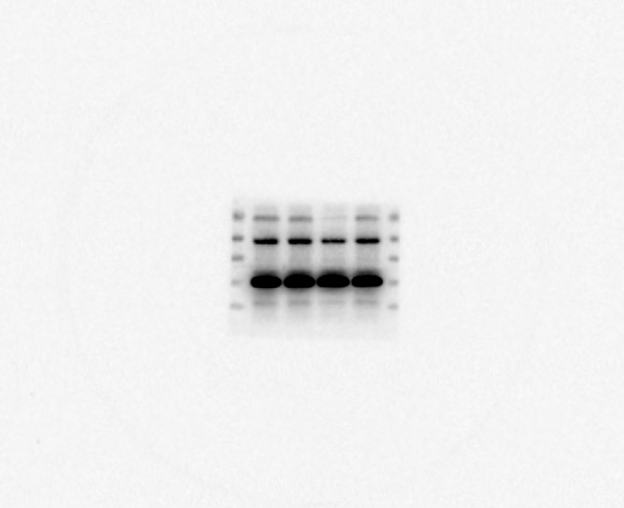
**

**Fig. 5g Occludin + β-actin**

**
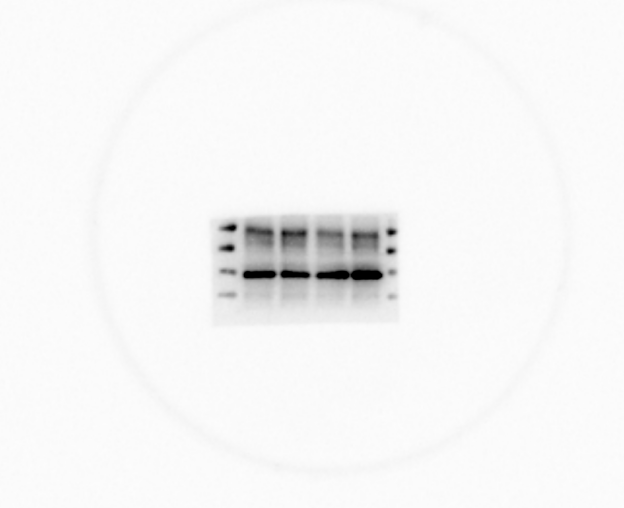

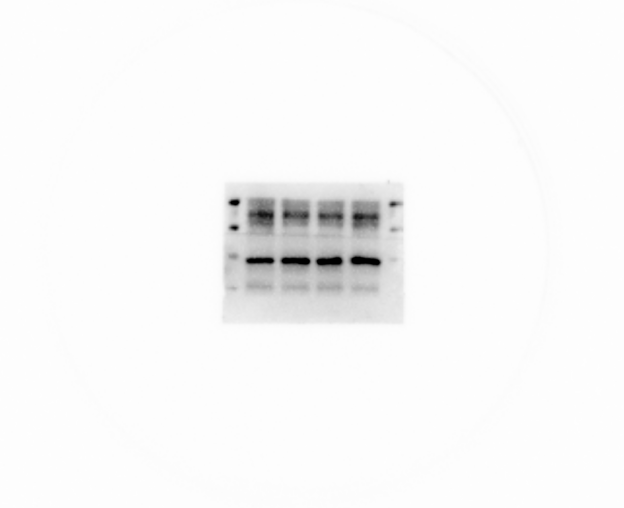
**

**
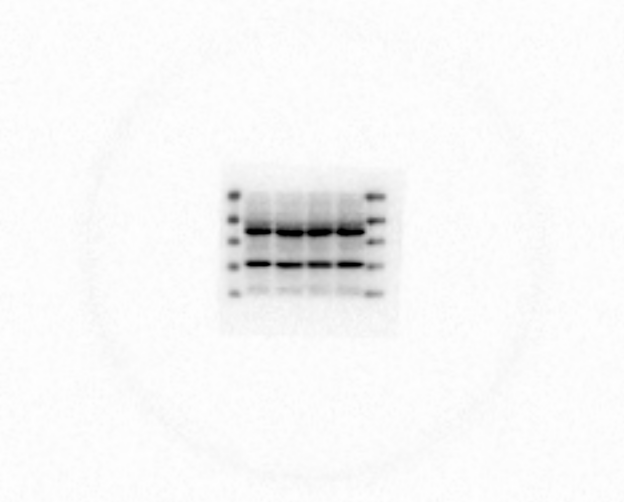

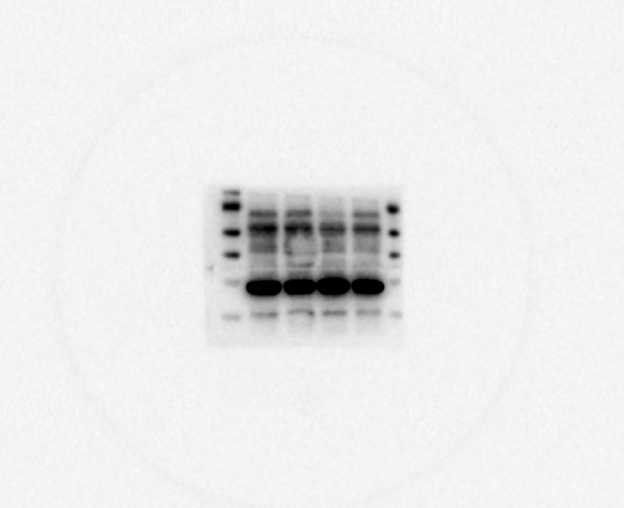

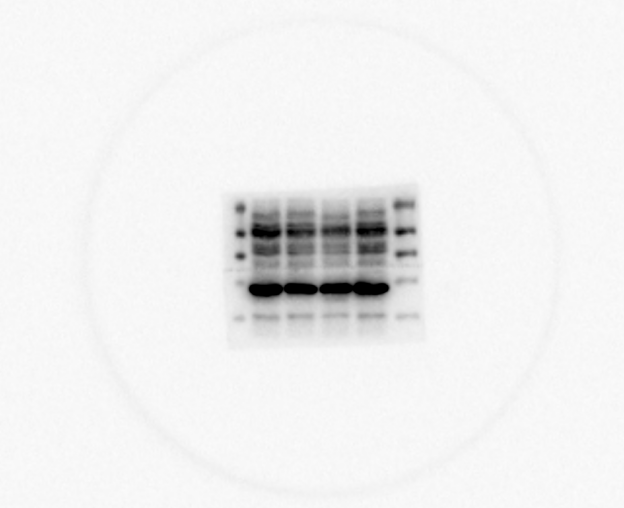
**

**Fig. 6a ROCK1 + β-actin**

**
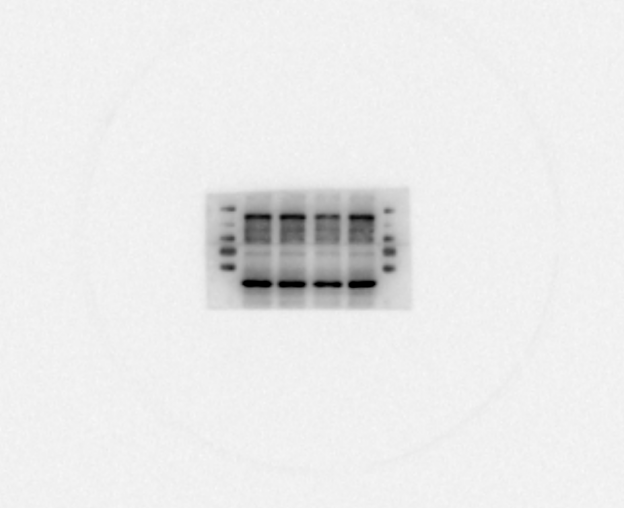

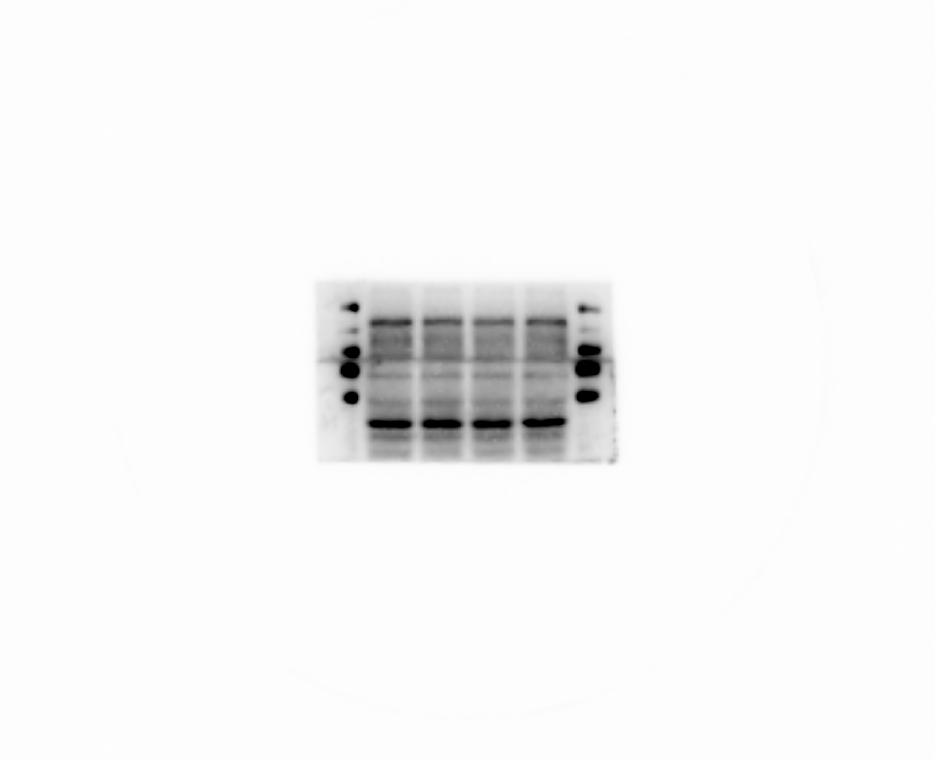
**

**
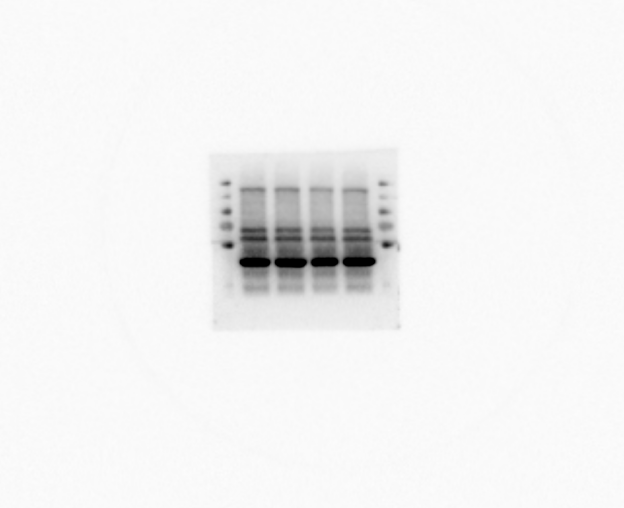

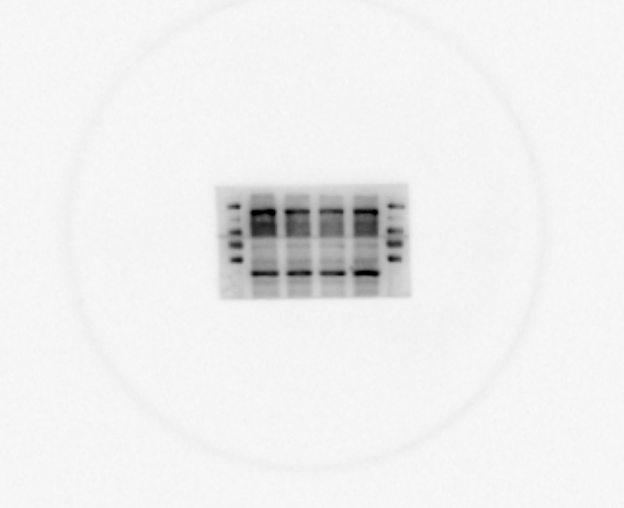
**

**
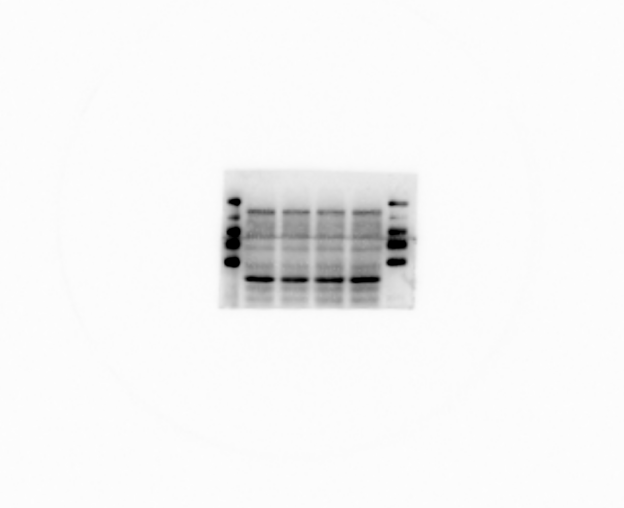
**

**Fig. 6b pMLC/MLC + β-actin**

1. **pMLC + β-actin**

1. **MLC + β-actin**

**Fig. 6c NMMHC IIA + β-actin**

**Fig. 7a ROCK1 + β-actin**

**Fig. 7b pMLC/MLC + β-actin**

1. **pMLC + β-actin**

1. **MLC + β-actin**

**Fig. 7c NMMHC IIA + β-actin**

**Fig. 7f NMMHC IIA + F-actin (Co-IP)**

**Fig. 7g ZO-1 + α-Tubulin**

**Fig. 7h Occludin + β-actin**

**Supplementary Fig. S1f ZO-1 + α-Tubulin**

**Supplementary Fig. S1g Occludin + β-actin**

**Supplementary Fig. S2f ZO-1 + α-Tubulin**

**Supplementary Fig. S2g Occludin + β-actin**

**Supplementary Fig. S2h ROCK1 + β-actin**

**Supplementary Fig. S2i pMLC/MLC + β-actin**

1. **pMLC + β-actin**

1. **MLC + β-actin**

**Supplementary Fig. S2j NMMHC IIA + β-actin**

**Supplementary Fig. S3a ROCK1 + β-actin**

**Supplementary Fig. S3b pMLC/MLC + β-actin**

1. **pMLC + β-actin**

1. **MLC + β-actin**

**Supplementary Fig. S3c NMMHC IIA + β-actin**

**Supplementary Fig. S3e NMMHC IIA + F-actin (Co-IP)**

**Supplementary Fig. S3f ZO-1 + α-Tubulin**

**Supplementary Fig. S3g Occludin + β-actin**

**Supplementary Fig. S4b NMMHC IIA + β-actin**

**Supplementary Fig. S5a ROCK1 + β-actin**

**Supplementary Fig. S5b pMLC/MLC + β-actin**

1. **pMLC + β-actin**

1. **MLC + β-actin**

**Supplementary Fig. S5c NMMHC IIA + β-actin**

**Supplementary Fig. S5e NMMHC IIA + F-actin (Co-IP)**

**Supplementary Fig. S5g ZO-1 + α-Tubulin**

**Supplementary Fig. S5h Occludin + β-actin**

**Supplementary Fig. S8a Claudin-5 + β-actin**

**Supplementary Fig. S8b Claudin-5 + β-actin**
