# Supplementary material for: The role of ROCK1/MLC/NMMHC IIA-actin signaling in ischemic stroke-induced blood-brain barrier disruption: implications for therapeutic intervention
Source: Cell Mol Life Sci. 2025 Oct 30;82(1):373. doi: 10.1007/s00018-025-05808-4 (PMC12575896; doi:10.1007/s00018-025-05808-4)
Supplement: Supplementary file 2 — Supplementary Material 2 (DOCX 109 MB) [file 18_2025_5808_MOESM2_ESM.docx]

**Supplementary Material**

**Supplementary Fig. S1 Endothelial conditional knockdown of NMMHC IIA (*Myh9^ECKD^*) rescues MCAO/R-induced cerebral ischemia-reperfusion injury in mice.** Mice received tamoxifen (150 mg/kg) for 5 consecutive days, followed by 1 h of ischemia and 24 h of reperfusion. **a** Representative brain sections stained with 2, 3, 5-triphenyl tetrazolium chloride (TTC), where infarcted areas appear white and non-infarcted regions red. **b** Quantitative analysis of infarct volume was performed for all experimental groups. **c** Representative neurological deficit scores, where higher scores correspond to more severe impairments. **d, e** Representative gross images of Evans Blue(EB)-stained brains, along with a quantitative assessment of EB extravasation (μg). **f, g** Representative Western blot data and quantitative analysis of the expression levels of the tight junction proteins ZO-1 and occludin. Data are presented as mean ± SD, n = 5. Statistical significance is denoted by NS (non-significant), ^##^*P* < 0.01 vs. *Myh9^fl/fl^* group, ^*^*P* < 0.05, ^**^*P* < 0.01 vs. *Myh9^fl/fl^* + MCAO/R group.

**Supplementary Fig. S2 Protective effects of blebbistatin on MCAO/R-induced ROCK/MLC/NMMHC IIA-actin activation in mice.** Mice were subjected to 1 h of ischemia and 24 h of reperfusion. Blebbistatin (10 mg/kg, i.p.) was administered 1 h after ischemia. **a** Representative brain sections stained with TTC, where infarcted areas appear white and non-infarcted regions red. **b** Quantitative analysis of infarct volume was performed for all experimental groups. **c** Representative neurological deficit scores, where higher scores correspond to more severe impairments. **d, e** Representative gross images of EB-stained brains, along with a quantitative assessment of EB extravasation (μg). **f, g** Representative Western blot data and quantitative analysis of the expression levels of the tight junction proteins ZO-1 and occludin. **h, i, j** Representative Western blot images and quantitative analyses of ROCK1, phosphorylated MLC (p-MLC), total MLC, and NMMHC IIA expression. Data are expressed as mean ± SD, with n = 5. Statistical significance is denoted by NS (non-significant), ^##^*P* < 0.01 compared to the Sham group, and ^*^*P* < 0.05, ^**^*P* < 0.01 compared to the MCAO/R group.

**Supplementary Fig. S3 Protective effects of blebbistatin on OGD/R-induced ROCK/MLC/NMMHC IIA-actin activation in bEnd.3 cells.** bEnd.3 cells were subjected to OGD/R and treated with blebbistatin (1 μM) for 18 h after OGD. **a, b, c** Representative Western blot data and quantitative analyses of ROCK1, p-MLC, MLC, and NMMHC IIA expression. **d** Representative immunofluorescence images of NMMHC IIA (green) and F-actin (red), with nuclei stained by DAPI in blue. **e** The results of co-immunoprecipitation (Co-IP) experiments, showing the interaction between NMMHC IIA and F-actin under OGD/R conditions. **f, g** Representative Western blot data and quantitative analyses of ZO-1 and occludin expression. Data are represented as mean ± SD, n = 5. Statistical significance is denoted by NS (non-significant), ^##^*P* < 0.01 versus the Control group, and ^**^*P* < 0.01 versus the OGD/R group. Scale bar, 50 μm.

**Supplementary Fig. S4 Identification of overexpression efficiency of NMMHC IIA in brain endothelium. a** Tail genomic DNA of *Myh9^iECKI^* mice was used for PCR genotyping according to corresponding primers. **b** Representative immunoblots and quantitative determinations of NMMHC IIA levels in mice. Data are represented as mean ± SD, n = 6.

**Supplementary Fig. S5 Protective effects of Y-27632 on OGD/R-induced ROCK/MLC/NMMHC IIA-actin activation in bEnd.3 cells.** bEnd.3 cells were subjected to OGD/R. Cells were treated with Y-27632 (1 μM) for 18 h after OGD. **a, b, c** Western blot data and quantitative analyses of ROCK1, p-MLC, MLC, and NMMHC IIA expression. **d** Representative immunofluorescence images of ROCK1 (green) and CD31 (red), with DAPI-stained nuclei in blue. **e** Representative immunofluorescence images of NMMHC IIA (green) and F-actin (red), with nuclei stained by DAPI in blue. **f** The results of Co-IP experiments, showing the interaction between NMMHC IIA and F-actin under OGD/R conditions. **g, h** Representative Western blot data and quantitative analyses of ZO-1 and occludin expression. Data are represented as mean ± SD, n = 5. Statistical significance is indicated as NS (non-significant), ^##^*P* < 0.01 versus the Control group, and ^*^*P* < 0.05, ^**^*P* < 0.01 versus the OGD/R group. Scale bar, 50 μm.

**Supplementary Fig. S6 Inhibition of NMMHC IIA and ROCK1 reduced cerebral vascular permeability after ischemia-reperfusion.** **a** Confocal microscopic images of the cerebral. FITC-dextran (40 kDa) was injected in left ventricle 24 h after ischemia-reperfusion. **b** Quantitative analysis was performed on values for FITC-dextran mean intensity of cerebral in mice. Data are presented as mean ± SD, n = 5. Statistical significance is denoted by NS (non-significant), *^##^P* < 0.01 vs. Sham group, ***P* < 0.01 vs. MCAO/R group.

**Supplementary Fig. S7 Inhibition of ROCK1, NMMHC IIA, and NMMHC IIA-actin interaction reduced the ROCK activity in bEnd.3 after OGD/R.** Lysates were collected from bEnd.3 cells in RIPA buffer with 1% PMSF and 2% phosphatase inhibitors. Protein (10 μg) was used in duplicate to determine ROCK activity following the manufacturer’s instructions (CSA001, Millipore). Data are presented as mean ± SD, n = 5. Statistical significance is denoted by NS (non-significant), *^##^P* < 0.01 vs. Control group, ***P* < 0.01 vs. OGD/R group.

**Supplementary Fig. S8 Inhibition of NMMHC IIA and ROCK1 increased the expression of Claudin-5 in cerebral brain tissue after ischemia-reperfusion. a** Western blot data and quantitative analysis of the expression levels of Claudin-5 in *Myh9^ECKD^* mice. **b** Western blot data and quantitative analysis of the expression levels of Claudin-5 in MCAO/R mice after administration of Y-27632. Data are presented as mean ± SD, n = 5. Statistical significance is denoted by NS (non-significant), *^##^P* < 0.01 vs. *Myh9^fl/fl^* or Sham group, ***P* < 0.01 vs. *Myh9^fl/fl^* + MCAO/R or MCAO/R group.

**Supplementary Table 1. Genotyping primers for *Myh9^ECKD^* and *Myh9^iECKI^* mice**

| **The following primers were used for genotyping of *Myh9^ECKD^* mice:** | | |
| --- | --- | --- |
|  | **Forward** | **Reverse** |
| *Myh9* | 5’-AAGACATGTCATTAGTGTCCTGAGAGCAGTG-3’ | 5’-TATGGCAGATTCAGGCATGGAGTTTGTGATG-3’ |
| *Myh9-*null | 5’-CGGTTCTGTCTTGCTACCAG-3’ | 5’-CAGGCATGGAGTTTGTGATG-3’ |
| Cre-KT | 5’-TGCCACGACCAAGTGACAGCAATG-3’ | 5’-ACCAGAGACGGAAATCCATCGCTC-3’ |
| **The following primers were used for genotyping of *Myh9^iECKI^* mice:** | | |
|  | **Forward** | **Reverse** |
| *Myh9*-loxp-3t | 5’-CCGGAACCATAACTTCGTATAGCA-3’ | 5’-CTCTACCAGCTCTACAATGGCCTC-3’ |
| *Myh9*-loxp-5t | 5’-TTATGGTAATCGTGCGAGAGGG-3’ | 5’-CACCTTGGAGAACTTGGGTG-3’ |
| H11-t | 5’-GGGCAGTCTGGTACTTCCAAGCT-3’ | 5’-ATATCCCCTTGTTCCCTTTCTGC-3’ |
| Cre-KT | 5’-TGCCACGACCAAGTGACAGCAATG-3’ | 5’-ACCAGAGACGGAAATCCATCGCTC-3’ |
